# Supplementary material for: Characteristics of clinician input in Canadian funding decisions for cancer drugs: a cross-sectional study based on CADTH reimbursement recommendations
Source: BMJ Open. 2023 Oct 16;13(10):e066378. doi: 10.1136/bmjopen-2022-066378 (PMC10583055; doi:10.1136/bmjopen-2022-066378)
Supplement: Author's manuscript [file bmjopen-2022-066378.draft_revisions.pdf]

BMJ Open

BMJ Open is committed to open peer review. As part of this commitment we make the peer review history of every article we publish publicly available.

When an article is published we post the peer reviewers' comments and the authors' responses online. We also post the versions of the paper that were used during peer review. These are the versions that the peer review comments apply to.

The versions of the paper that follow are the versions that were submitted during the peer review process. They are not the versions of record or the final published versions. They should not be cited or distributed as the published version of this manuscript.

BMJ Open is an open access journal and the full, final, typeset and author-corrected version of record of the manuscript is available on our site with no access controls, subscription charges or pay-per-view fees (http://bmjopen.bmj.com).

If you have any questions on BMJ Open's open peer review process please email info.bmjopen@bmj.com

BMJ Open

Association of clinician input and quality of evidence supporting cancer drug funding decisions in Canada

Journal:	*BMJ Open*
Manuscript ID	bmjopen-2022-066378
Article Type:	Original research
Date Submitted by the Author:	06-Jul-2022

Complete List of Authors:	Jenei, Kristina; The University of British Columbia, School of Population and Public Health<br>Meyers, Daniel E.; University of Calgary, Department of Medicine<br>Prasad, Vinay; University of California San Francisco, Department of Epidemiology and Biostatistics

Keywords:	Health policy < HEALTH SERVICES ADMINISTRATION & MANAGEMENT, HEALTH ECONOMICS, ONCOLOGY

SCHOLARONE™
Manuscripts

I, the Submitting Author has the right to grant and does grant on behalf of all authors of the Work (as defined in the below author licence), an exclusive licence and/or a non-exclusive licence for contributions from authors who are: i) UK Crown employees; ii) where BMJ has agreed a CC-BY licence shall apply, and/or iii) in accordance with the terms applicable for US Federal Government officers or employees acting as part of their official duties; on a worldwide, perpetual, irrevocable, royalty-free basis to BMJ Publishing Group Ltd ("BMJ") its licensees and where the relevant Journal is co-owned by BMJ to the co-owners of the Journal, to publish the Work in this journal and any other BMJ products and to exploit all rights, as set out in our licence.

The Submitting Author accepts and understands that any supply made under these terms is made by BMJ to the Submitting Author unless you are acting as an employee on behalf of your employer or a postgraduate student of an affiliated institution which is paying any applicable article publishing charge ("APC") for Open Access articles. Where the Submitting Author wishes to make the Work available on an Open Access basis (and intends to pay the relevant APC), the terms of reuse of such Open Access shall be governed by a Creative Commons licence – details of these licences and which Creative Commons licence will apply to this Work are set out in our licence referred to above.

Other than as permitted in any relevant BMJ Author's Self Archiving Policies, I confirm this Work has not been accepted for publication elsewhere, is not being considered for publication elsewhere and does not duplicate material already published. I confirm all authors consent to publication of this Work and authorise the granting of this licence.

For peer review only

Association of clinician input and quality of evidence supporting cancer drug funding decisions in Canada

Kristina Jenei BSN MSc[1], Daniel E. Meyers MD MSc[2], Vinay Prasad MD, MPH[1]

[1] School of Population and Public Health, University of British Columbia, Vancouver, British Columbia, Canada
[2] Department of Medicine, University of Calgary, Calgary, Canada
[3] Department of Epidemiology and Biostatistics, University of California San Francisco, United States

For peer review only

Word count (text): 2130
Tables: 3
Figure: 1

Corresponding author:
Kristina Jenei, BSN MSc
Address: 2206 East Mall
Vancouver, British Columbia, Canada
V6T 1Z3
Telephone: 1-778-239-2163
Email: kjenei@mail.ubc.ca

For peer review only - http://bmjopen.bmj.com/site/about/guidelines.xhtml

Abstract

Objective: To examine characteristics of clinician input to the pan-Canadian Oncology Drug Review (pCODR) for cancer drug funding recommendations from 2016 to 2020.

Design, Setting, and Participants: Retrospective cohort study included 62 reimbursement decisions from pCODR from 2016 to 2020.

Interventions: pCODR recommendations were analyzed for the number of clinicians consulted on each submission, affiliation, number of submissions per clinician, declared financial conflict of interests (FCOIs), randomization, type of masking, primary endpoint, study phase, and whether the study demonstrated improvement in overall survival (OS) and progression free survival (PFS).

Main outcome measures: The main outcome was clinician support for the initial funding recommendation.

Results: The study consisted of 62 submissions, in which 48 included clinician input. A total of 129 clinicians provided 342 consultations. The majority (59%) provided input on less than five submissions, however, a small proportion (4%) consulted on over 10. Nearly all clinicians were physicians (125; 96%). From the 342 consultations, 228 declared financial conflicts (67%). The most common conflicts were payments for advisory roles (51%) and honorariums (23%) with the sponsoring company. Of the 48 cancer drugs under review, clinicians recommended funding 46 (96%). Only 12 (*) demonstrated substantial benefit, according to the ESMO-MCBS score. Drugs recommended for funding were more likely to have improved PFS and OS data. However, most cancer drugs supported by clinicians demonstrated no change in health-related quality of life (HRQoL), including one that demonstrated worsened HRQoL.

Conclusion: Clinicians offer crucial information in funding decisions. However, we found clinicians strongly supported funding nearly all cancer drugs under review, despite most not offering substantial benefit to patients nor gains in quality of life. While these drugs might be helpful options in clinical practice, funding numerous cancer drugs is unsustainable for public health systems.

For peer review only - http://bmjopen.bmj.com/site/about/guidelines.xhtml

Strengths and Limitations

- The study is the first to examine the association between clinician funding recommendations and the evidence underpinning cancer drug reimbursement decisions
- The study used a homogenous sample of solid tumour indications to assess clinical benefit, however, cannot be generalised to all submissions
- We assessed the strength of evidence underpinning clinician funding support
- Clinician submissions were made collectively and cannot be attributed to individual views
- The financial conflict of interests are self-reported data by clinicians

For peer review only

Keywords
Health technology assessment; oncology; drug approvals; health policy; conflict of interest

For peer review only

For peer review only - http://bmjopen.bmj.com/site/about/guidelines.xhtml

Introduction

The Canadian Agency for Drugs and Technology in Health (CADTH) guides funding recommendations for Canadian provinces. CADTH, and its oncology assessment arm, the pan-Canadian Oncology Drug Review (pCODR)[1], evaluates medicines through a deliberative framework that includes clinical and economic evidence, clinician, and patient feedback.[2] While there are special exemptions, only medicines that are funded by the province are accessible to patients. Once a drug is reviewed, the pCODR Expert Review Committee (pERC)[3] — an appointed interdisciplinary body comprised of clinicians, ethicists, health economists, and patients — provide the final reimbursement recommendation. While each Canadian province independently decides whether a drug will be funded under their public drug plans, provinces typically follow CADTH recommendations.[4]

Clinicians are involved in Canadian cancer drug funding decisions through two mechanisms. First, every drug review team includes oncologists to develop the systematic review protocol, interpret and provide real-world applicability for the study results. Once pERC reviews the clinical and economic reports and provides an initial funding recommendation, there is a second opportunity for clinician feedback. Since October 2016, CADTH provides an opportunity for additional feedback through external "calls for clinician input". Registered clinicians, such as oncologists, pharmacists, and oncology nurses can submit feedback. While submissions can be made individually, CADTH encourages clinicians to submit in a group or health care association. Each submission must include an oncologist. This feedback is used to determine the final funding recommendation and published on the pCODR website.

Given clinical trials are not always generalizable to the broader population, clinician and patient perspectives are important to understand local values of preferences. However, previous research exploring the role of clinicians in Canadian funding decisions found widespread financial conflict of interests (FCOIs).[5] In the United States (US), FCOIs are widespread in oncology[6] and have been found to influence prescribing patterns, formulary recommendations, research, and recommendations for clinical practice guidelines.[7] Given these associations, we sought to evaluate characteristics of clinician input beyond FCOIs, including the association with funding support and underlying clinical evidence for the cancer drugs under review.

For peer review only - http://bmjopen.bmj.com/site/about/guidelines.xhtml

Methods

Setting

We conducted a retrospective cohort study of reimbursement recommendations to pCODR for solid tumour indications between 2016 and 2020. The study examined publicly available data and therefore was exempt from institutional review board approval, in accordance with the US Department of Health and Human Services, 45 CFR §46.102(f). We adhered to the Strengthening the Reporting of Observational studies in Epidemiology (STROBE) reporting guidelines.

Source of data and variables

A systematic search of the pCODR website was completed to identify characteristics of physician submissions and supporting evidence. We used a sample of solid tumor indications from an existing dataset.[8] Supportive care medicines, hematologic neoplasms, pediatric indications, and biosimilars were excluded. By focusing on solid tumor indications, the dataset was homogenous which allowed us to assess the clinical benefit using the European Society for Medical Oncology Magnitude of Clinical Benefit Scale (ESMO-MCBS)[9], which was designed for solid tumour indications. We extracted the name of the drug, date and year of reimbursement recommendation and supporting clinical trial information, such as randomization, masking, overall survival and progression free survival outcomes, health related quality of life data, and estimated price (per 28-day cycle). While final drug prices are confidentially negotiated with the manufacturer after the funding recommendation, estimated prices are included in the pCODR economic evaluation reports. For more information on how these data were collected and how the ESMO-MCBS scores were calculated, see the previous publication.[8]

Clinician input on cancer drug submissions

In addition to the clinical evidence supporting each drug, we extracted data related to the clinician submissions. These data included the number of clinicians consulted on each submission, affiliation, number of submissions per clinician, and declared FCOIs. Clinicians provide feedback on the initial funding recommendation. On the submission forms, clinicians are asked whether they "agree", "disagree", or "agree in part" with the initial funding recommendation. With this feedback, pCODR can revise the final funding recommendation. While there are often numerous clinicians involved in each drug review, the feedback form is submitted collectively. When the clinicians agreed with a positive funding recommendation or disagreed with a negative funding recommendation, we coded this as "fund". When the clinicians agreed with a negative recommendation or disagreed with a positive funding recommendation, we coded this as "do not fund". When the clinician feedback was to "agree in part", we reviewed comments in the feedback documents to ensure coding was accurate.

Conflict of interest data

Despite one collective feedback form, FCOI forms are submitted individually. FCOI forms require clinicians to disclose whether they have received payments over the previous two years from any company or organization that may have direct or indirect

For peer review only - http://bmjopen.bmj.com/site/about/guidelines.xhtml

interest in the drug under review. The forms require disclosure on the type of activity the payment was for (e.g., advisory board, conferences, honoraria, research, sponsorship of events, operations, travel, among others). Clinicians must also provide the names of the manufacturers who they have received funds from, including whether these payments exceeded $10,000 and whether they have personal or commercial relationships with any interest group in relation to the drug under submission. Information related to all these categories were also extracted.

Patient and Public Involvement

No patients nor publics were involved.

For peer review only

Results
Overall, there were 62 drug funding reviews by CADTH for solid tumour indications between 2016 and 2020. Of these, clinician input was available for 48 submissions. For 14, there were no clinician feedback published on the pCODR website (there are no reasons provided for why these submissions did not have clinician input).

Table 1 reports the descriptive characteristics of clinicians that were consulted during cancer drug funding decisions. On average, 4 (IQR: 2-8) clinicians provided feedback per drug. In the 48 drug reviews, there was 342 submissions from clinicians for feedback on funding recommendations. However, there were only 129 unique clinicians which suggests clinicians often consulted on more than one drug. Of these, 124 (96%) were physicians and 5 (4%) were oncology pharmacists. Most clinicians (76; 59%) consulted on only one drug review. However, a small minority (4; 3%) made repeat submissions on more than 10 drugs.

Table 1. Characteristics of clinicians that submitted input to the pan-Canadian Oncology Drug Review

**Characteristic**	
Reimbursement decisions (2016-2020)	62 (100)
Reimbursement decisions with clinician submissions	48 (100)
Clinician recommendation	
Fund	46 (96)
Do not fund	2 (4)
Median number of clinicians that offered feedback per drug review (IQR)	4 (2-8)
Total number of clinician feedback submissions	342 (100)
Number of funding decisions consulted	
One	76
Two	16
3 to 10	33 ()
More than 10	4 (3)
Unique clinicians	129 (100)
Affiliation	
Physician	124 (96)
Oncology pharmacist	5 (4)

For peer review only

For peer review only - http://bmjopen.bmj.com/site/about/guidelines.xhtml

We found numerous financial conflicts of interests **(Figure 1)**. Of the 324 instances where clinicians were consulted during the drug review, 228 (67%) reported FCOIs **(Table 2)**. Of these, 149 (44%) were with the manufacturer of the drug under review. The average number of conflicts a clinician disclosed was 3 (IQR: 2 to 6). Advisory roles were the most common type of conflict (166; 51%), followed by honorariums (23%), payments to attend conferences, research, and sponsorship of events (7%, respectively). We found 9 documents (3%) that were incomplete.

Table 2. Financial conflict of interests among clinicians consulted on drug funding decisions

Characteristic	Submissions (n=342)
Clinician submissions declaring conflicts	228 (67)
Clinician submissions with conflicts with the manufacturer of the drug under review	149 (44)
Avg number of conflicts per submission	3 (2-6)
Types of conflicts reported	
Advisory role	166 (51)
Honoraria	74 (23)
Conference	22 (7)
Research	22 (7)
Events	23 (7)
Operations	2 (1)
Travel	3 (1)
Speaking	12 (1)
Incomplete COI documentation	9 (3)

Most clinicians were in favor of funding the drug under review (96%). **Table 3** reports the association between clinician recommendation and supporting evidence. Of the 48 submissions that included clinician input, 46 (96%) had positive clinician feedback to fund the drug. This included 12 (26%) drugs that received negative recommendations from CADTH in which clinicians submitted feedback in support of funding the drug. Only 2 (4%) submissions believed the drug should not be funded.

For peer review only - http://bmjopen.bmj.com/site/about/guidelines.xhtml

Table 3. Association between clinician funding recommendation and supporting evidence

	Clinician recommendation (n=48)	
	Fund (n=46)	Do not fund (n=2)
CADTH decision		
Positive	29 (63)	0 (0)
Negative	12 (26)	2 (100)
Submission w/conflicts		
Yes	44 (96)	1 (50)
No	2 (4)	1 (50)
Submissions w/ conflicts with manufacturer		
Yes	34 (74)	1 (50)
No	12 (26)	1 (50)
ESMO-MCBS Score		
Substantial benefit	14 (30)	2 (100)
No substantial benefit	32 (70)	0 (0)
RCT		
Yes	34 (74)	2 (100)
No	12 (26)	0 (0)
Phase		
1	3 (7)	0 (0)
2	10 (22)	0 (0)
3	33 (72)	2 (100)
Masked studies		
Yes	20 (43)	2 (100)
No	14 (30)	0 (0)
NR	12 (26)	0 (0)
OS		
Improved	18 (39)	0 (0)
Not improved	12 (26)	1 (50)
NA	16 (35)	1 (50)
PFS Data		
Improved	25 (54)	0 (0)
Not improved	5 (11)	0 (0)
NA	16 (35)	2 (100)
HRQoL		
Improved	5 (11)	0 (0)
No change	32 (70)	2 (100)
Worsen	1 (2)	0 (0)
NA	5 (11)	0 (0)
Price (per 28 cycle)		
Under $5000	5 (11)	0 (0)
$5000 to $9,999	27 (59)	1 (50)
Over $10,000	14 (30)	1 (50)

For peer review only

For peer review only - http://bmjopen.bmj.com/site/about/guidelines.xhtml

Among drugs recommended for funding, most (32; 70%) did not offer substantial benefit, as per ESMO-MCBS. Clinicians recommended funding 18 (39%) drugs that had data that demonstrated an OS benefit, 12 (26%) without an OS benefit, and 16 (35%) that had no data on OS. Similarly, clinicians recommended funding 25 drugs (54%) that demonstrated an improved PFS benefit, 5 (11%) that did not improve PFS, and 16 drugs (35%) that did not have any PFS data. Nearly two thirds of drugs (32; 70%) that received funding support by clinicians demonstrated no change in HRQoL, with one that worsened HRQoL outcomes. Most cancer drugs that received clinician funding support were between $5000 to $9,999 per month (59%), including 14 (30%) that were over $10,000 per 28-day cycle (Table 3).

For peer review only

Discussion

We sought to examine characteristics of clinicians consulted in Canadian cancer drug funding decisions from 2016 to 2020. We examined the association between clinician drug funding recommendations and supporting evidence for the cancer drugs under review. Further, we explored the extent of FCOIs disclosed in clinician submissions. We found that clinicians who consult on Canadian funding decisions overwhelmingly support funding the cancer drug under review. Of the 48 drugs, 46 (96%) received supportive funding feedback from clinicians, even when most drugs did not offer substantial benefit to patients nor improved quality of life. These findings have important implications.

First, while clinician input is crucial to ensure real world clinical practice implications are included in reimbursement decisions, funding all cancer drugs is not possible for publicly funded health care systems. Our findings might highlight a mismatch of objectives between national health technology assessment (HTA) bodies and clinicians. The duty of a clinician to care for individual patients differs from HTA bodies who attempt to maximize population health outcomes for every dollar spent. For clinicians, it might be advantageous to have numerous treatment options to offer patients, including a spectrum of available medicines given differential responses to individual therapies. However, at the national reimbursement level, using numerous high-priced cancer drugs incurs enormous budget impacts and is unsustainable. Indeed, clinicians supported funding nearly all drugs priced over $5000 per 28-day cycle. This is especially concerning in oncology where many high-priced cancer drugs approved through regulatory agencies often only offer marginal benefit, if any at all.[8,10,11]

Second, all health systems must engage in rationing. Health care dollars are finite and difficult trade-offs must be made. Currently, Canada is third highest for total dollars spent on pharmaceuticals among 31 countries included in The Organisation for Economic Co-operation and Development (OECD).[12] In 2020, the largest proportion of Canadian public drug funding (22.9%) was spent on oncology agents.[13] In a previous study, we found that only half of cancer drugs that received a positive funding recommendation from pCODR had evidence of improved survival.[8] Similarly, the Patented Medicine Prices Review Board (PMPRB) —the federal agency responsible for regulating drug prices of new patented medicines in Canada — classified over 90% of new medicines as offering “moderate, little, or no improvement” in clinical benefit.[14]

Third, we found widespread conflicts of interests. Of the 342 submissions, 228 (67%) declared conflicts and nearly half (149; 44%) were directly with the manufacturer of the drug under review. While the relationship between clinician behaviour and FCOIs in Canada might warrant further investigation, we confirm previous research[5] that these findings are higher than FCOIs disclosed in US Food and Drug Administration Drug Advisory Committee meetings.[15] Previous Canadian research suggests FCOIs might influence clinician input and subsequent feedback influences the final recommendation. Lexchin found that 75% of clinicians who disagreed with a negative funding recommendation declared a conflict with the manufacturer under review.[5] However, in

For peer review only - http://bmjopen.bmj.com/site/about/guidelines.xhtml

the nine negative initial funding recommendations, pCODR changed their final recommendation once. Similarly, in our study, the initial funding recommendation was only changed once after stakeholder feedback. However, the literature about FCOIs and clinician behaviour suggests that there is a relationship between financial payments to clinicians and prescribing patterns, formulary recommendations, research, and recommendations for clinical practice guidelines.[6,7]

For peer review only

Strengths and Limitations

Our retrospective cohort study has strengths and limitations. It is unique as it is the first evaluation of characteristics of clinician input in Canadian funding decision that includes an assessment of the supporting evidence of the drugs under review. However, we encountered limitations. First, we were limited to a cohort of cancer drugs that was accepted and reviewed by CADTH. Given the opportunity, it is reasonable to assume many clinicians would prefer robust, mature evidence demonstrating improvements in patient-centered outcomes, such as overall survival and quality of life. Second, feedback forms where clinicians indicate their support for drug funding are submitted collectively, not individually. Therefore, clinician input cannot be attributed to individual views. For this reason, we examined collective feedback on submissions. Third, we are limited by a cohort of submissions for solid tumour indications which might differ compared to other malignancies. However, this approach allows for a homogenous dataset for which clinical benefit can be measured using the ESMO-MCBS. Lastly, we are limited by the publicly available data in pCODR reports. Given the complexity of evaluating evidence from oncology clinical trials, these reports might not offer insight into the nuanced discussions that likely occurred throughout the pCODR deliberations.

Conclusions

Clinicians offer crucial information in funding decisions. However, we found overwhelming support for nearly all drugs under review, even medicines that did not demonstrate improved OS benefit nor quality of life — two of the most important patient-centered outcomes. Further, we found numerous FCOIs. These findings raise questions about the role of stakeholder input in Canadian funding decisions and possible incongruity between the objectives of national health technology assessment bodies and practising clinicians.

For peer review only

Ethics approval: The study examined publicly available data and therefore was exempt from institutional review board approval, in accordance with the US Department of Health and Human Services, 45 CFR §46.102(f).

Contributor statement: Ms. Jenei lead the analysis, drafting, and dissemination. Drs. Meyers and Prasad were also involved in the drafting and editing of the manuscript. Dr. Prasad was responsible for supervision and conceptualization.

Competing interests: Dr. Prasad Disclosures: (Research funding) Arnold Ventures (Royalties) Johns Hopkins Press, Medscape, MedPage (Consulting) UnitedHealtcare. (Speaking fees) Evicore. New Century Health (Other) Plenary Session podcast has Patreon backers. Ms. Jenei and Dr. Meyers report no conflicts.

Funding statement: This study was funded by a grant from Arnold Ventures (Grant number: N/A). The funder was not involved in the manuscript.

Data sharing statement: Data are available upon reasonable request.

For peer review only

References

1. About the pan-Canadian Oncology Drug Review (pCODR) | CADTH.ca. 2019. https://cadth.ca/pcodr/about-pcodr. Accessed August 20, 2019.
2. CADTH. About CADTH. 2019. https://www.cadth.ca/about-cadth. Accessed July 17, 2019.
3. Health CA for D and T in. *PCODR Expert Review Committee Deliberative Framework*.; 2016. www.cadth.ca/pcodr. Accessed October 30, 2019.
4. Gamble JM, Weir DL, Johnson JA, Eurich DT. Analysis of drug coverage before and after the implementation of Canada's Common Drug Review. *CMAJ*. 2011;183(17):E1259. doi:10.1503/CMAJ.110670/-/DC1
5. Lexchin J. Financial conflicts of interest of clinicians making submissions to the pan-Canadian Oncology Drug Review: a descriptive study. *BMJ Open*. 2019;9:30750. doi:10.1136/bmjopen-2019-030750
6. Meyers DE, Chisamore TM, McInnes MDF, Gyawali B, Prasad V, Booth CM. Industry payments to US physicians for cancer therapeutics: An analysis of the 2016–2018 open payments datasets. *J Cancer Policy*. 2021;28:100283. doi:10.1016/J.JCPO.2021.100283
7. Mitchell AP, Trivedi NU, Gennarelli RL, et al. Are Financial Payments from the Pharmaceutical Industry Associated with Physician Prescribing? A Systematic Review. *Ann Intern Med*. 2021;174(3):353-361. doi:10.7326/M20-5665
8. Meyers DE, Jenei K, Chisamore TM, Gyawali B. Evaluation of the Clinical Benefit of Cancer Drugs Submitted for Reimbursement Recommendation Decisions in Canada. *JAMA Intern Med*. 2021;181(4):499-508. doi:10.1001/jamainternmed.2020.8588
9. Cherny NI, Dafni U, Bogaerts J, et al. ESMO-Magnitude of Clinical Benefit Scale version 1.1. *Ann Oncol*. 2017;28(10):2340-2366. doi:10.1093/ANNONC/MDX310
10. Fojo T, Mailankody S, Lo A. Unintended consequences of expensive cancer therapeutics - The pursuit of marginal indications and a me-too mentality that stifles innovation and creativity: The John Conley lecture. *JAMA Otolaryngol - Head Neck Surg*. 2014;140(12):1225-1236. doi:10.1001/jamaoto.2014.1570
11. Cherla A, Naci H, Kesselheim AS, Gyawali B, Mossialos E. Assessment of Coverage in England of Cancer Drugs Qualifying for US Food and Drug Administration Accelerated Approval. *JAMA Intern Med*. 2021;181(4):490-498. doi:10.1001/JAMAINTERNMED.2020.8441
12. Health resources - Pharmaceutical spending - OECD Data. https://data.oecd.org/healthres/pharmaceutical-spending.htm#indicator-chart. Accessed December 1, 2021.
13. Canadian Institute for Health Information. *Prescribed Drug Spending in Canada* .; 2020. https://secure.cihi.ca/free_products/prescribed-drug-spending-in-canada-2020-report-en.pdf. Accessed June 3, 2022.
14. Lexchin J. Canadian status of "drugs to avoid" in 2017: a descriptive analysis. *C Open*. 2018;6(3):E430. doi:10.9778/CMAJO.20180049
15. Lurie P, Almeida CM, Stine N, Stine AR, Wolfe SM. Financial Conflict of Interest Disclosure and Voting Patterns at Food and Drug Administration Drug Advisory Committee Meetings. *JAMA*. 2006;295(16):1921-1928. doi:10.1001/JAMA.295.16.1921

For peer review only - http://bmjopen.bmj.com/site/about/guidelines.xhtml

Figure. Proportion of disclosed financial conflicts by clinicians within each drug funding assessment (n=48)

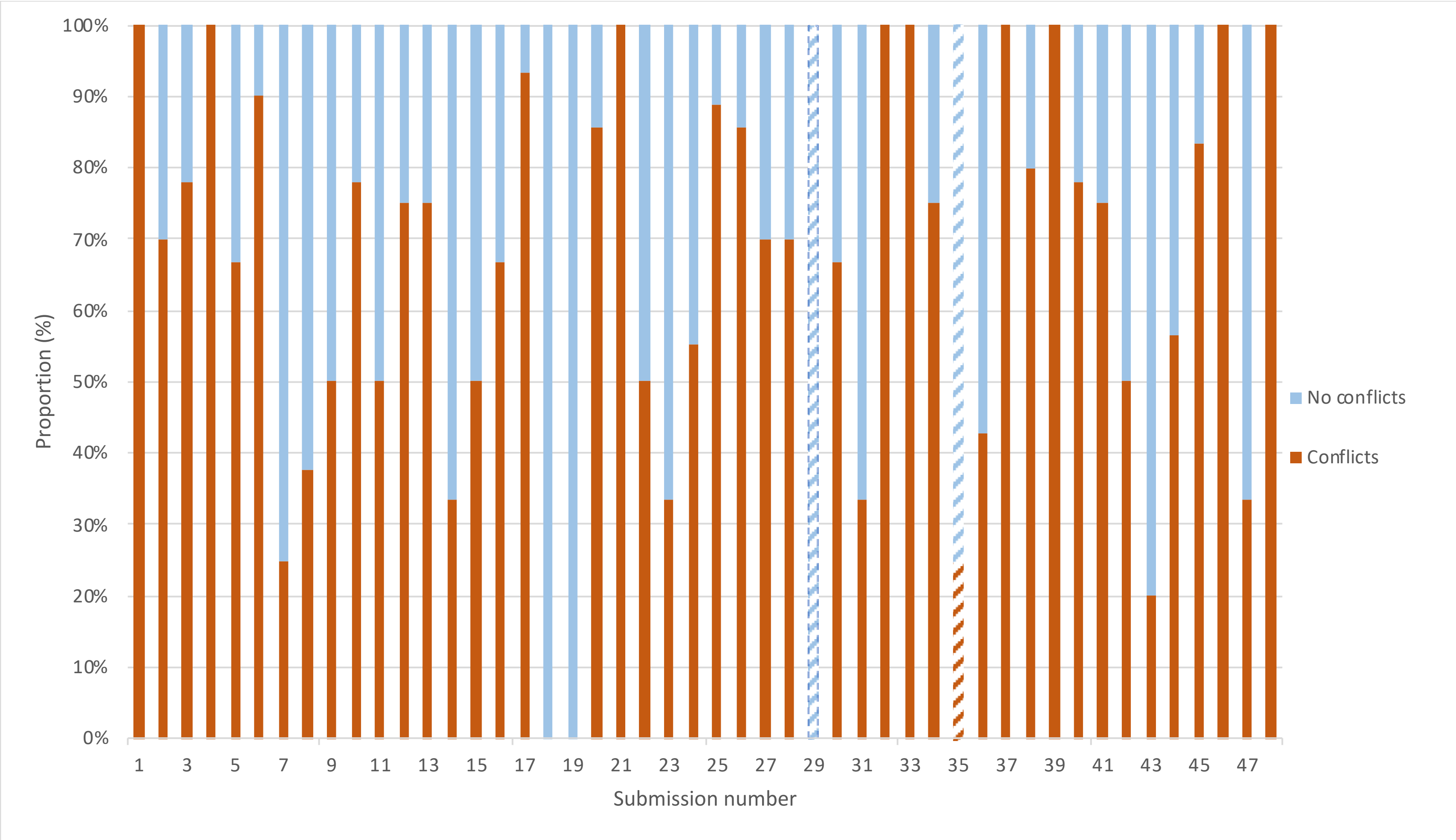

[a] The two reviews that received non-funding recommendations from clinicians are submission #29 and #35

STROBE Statement—Checklist of items that should be included in reports of ***cohort studies***

Characteristics of clinician input in Canadian funding decisions for cancer drugs

	Item No	Recommendation	Page No
**Title and abstract**	1	(*a*) Indicate the study's design with a commonly used term in the title or the abstract<br>(*b*) Provide in the abstract an informative and balanced summary of what was done and what was found	1-2
**Introduction**			
Background/rationale	2	Explain the scientific background and rationale for the investigation being reported	5
Objectives	3	State specific objectives, including any prespecified hypotheses	5
**Methods**			
Study design	4	Present key elements of study design early in the paper	6
Setting	5	Describe the setting, locations, and relevant dates, including periods of recruitment, exposure, follow-up, and data collection	6
Participants	6	(*a*) Give the eligibility criteria, and the sources and methods of selection of participants. Describe methods of follow-up<br>(*b*) For matched studies, give matching criteria and number of exposed and unexposed	N/A
Variables	7	Clearly define all outcomes, exposures, predictors, potential confounders, and effect modifiers. Give diagnostic criteria, if applicable	6
Data sources/ measurement	8*	For each variable of interest, give sources of data and details of methods of assessment (measurement). Describe comparability of assessment methods if there is more than one group	6
Bias	9	Describe any efforts to address potential sources of bias	NA
Study size	10	Explain how the study size was arrived at	6
Quantitative variables	11	Explain how quantitative variables were handled in the analyses. If applicable, describe which groupings were chosen and why	6
Statistical methods	12	(*a*) Describe all statistical methods, including those used to control for confounding<br>(*b*) Describe any methods used to examine subgroups and interactions<br>(*c*) Explain how missing data were addressed<br>(*d*) If applicable, explain how loss to follow-up was addressed<br>(*e*) Describe any sensitivity analyses	NA (descriptive Page 6)
**Results**			
Participants	13*	(a) Report numbers of individuals at each stage of study—eg numbers potentially eligible, examined for eligibility, confirmed eligible, included in the study, completing follow-up, and analysed<br>(b) Give reasons for non-participation at each stage<br>(c) Consider use of a flow diagram	8
Descriptive data	14*	(a) Give characteristics of study participants (eg demographic, clinical, social) and information on exposures and potential confounders<br>(b) Indicate number of participants with missing data for each variable of interest	8

For peer review only - http://bmjopen.bmj.com/site/about/guidelines.xhtml

		(c) Summarise follow-up time (eg, average and total amount)	
Outcome data	15*	Report numbers of outcome events or summary measures over time	8

For peer review only

Main results	16	(*a*) Give unadjusted estimates and, if applicable, confounder-adjusted estimates and their precision (eg, 95% confidence interval). Make clear which confounders were adjusted for and why they were included<br>(*b*) Report category boundaries when continuous variables were categorized<br>(*c*) If relevant, consider translating estimates of relative risk into absolute risk for a meaningful time period	8
Other analyses	17	Report other analyses done—eg analyses of subgroups and interactions, and sensitivity analyses	NA
**Discussion**			
Key results	18	Summarise key results with reference to study objectives	
Limitations	19	Discuss limitations of the study, taking into account sources of potential bias or imprecision. Discuss both direction and magnitude of any potential bias	9
Interpretation	20	Give a cautious overall interpretation of results considering objectives, limitations, multiplicity of analyses, results from similar studies, and other relevant evidence	9
Generalisability	21	Discuss the generalisability (external validity) of the study results	9/10
**Other information**			
Funding	22	Give the source of funding and the role of the funders for the present study and, if applicable, for the original study on which the present article is based	1

*Give information separately for exposed and unexposed groups.

Note: An Explanation and Elaboration article discusses each checklist item and gives methodological background and published examples of transparent reporting. The STROBE checklist is best used in conjunction with this article (freely available on the Web sites of PLoS Medicine at http://www.plosmedicine.org/, Annals of Internal Medicine at http://www.annals.org/, and Epidemiology at http://www.epidem.com/). Information on the STROBE Initiative is available at http://www.strobe-statement.org.

For peer review only

BMJ Open

Characteristics of clinician input in Canadian funding decisions for cancer drugs: a cross-sectional, descriptive study based on CADTH reimbursement recommendations

Journal:	*BMJ Open*
Manuscript ID	bmjopen-2022-066378.R1
Article Type:	Original research
Date Submitted by the Author:	06-Jun-2023
Complete List of Authors:	Jenei, Kristina ; The London School of Economics and Political Science, Department of Health Policy<br>Meyers, Daniel E.; University of Calgary, Department of Medicine
**Primary Subject Heading**:	Health economics
Secondary Subject Heading:	Oncology, Evidence based practice
Keywords:	Health policy < HEALTH SERVICES ADMINISTRATION & MANAGEMENT, HEALTH ECONOMICS, ONCOLOGY

SCHOLARONE™
Manuscripts

I, the Submitting Author has the right to grant and does grant on behalf of all authors of the Work (as defined in the below author licence), an exclusive licence and/or a non-exclusive licence for contributions from authors who are: i) UK Crown employees; ii) where BMJ has agreed a CC-BY licence shall apply, and/or iii) in accordance with the terms applicable for US Federal Government officers or employees acting as part of their official duties; on a worldwide, perpetual, irrevocable, royalty-free basis to BMJ Publishing Group Ltd ("BMJ") its licensees and where the relevant Journal is co-owned by BMJ to the co-owners of the Journal, to publish the Work in this journal and any other BMJ products and to exploit all rights, as set out in our licence.

The Submitting Author accepts and understands that any supply made under these terms is made by BMJ to the Submitting Author unless you are acting as an employee on behalf of your employer or a postgraduate student of an affiliated institution which is paying any applicable article publishing charge ("APC") for Open Access articles. Where the Submitting Author wishes to make the Work available on an Open Access basis (and intends to pay the relevant APC), the terms of reuse of such Open Access shall be governed by a Creative Commons licence – details of these licences and which Creative Commons licence will apply to this Work are set out in our licence referred to above.

Other than as permitted in any relevant BMJ Author's Self Archiving Policies, I confirm this Work has not been accepted for publication elsewhere, is not being considered for publication elsewhere and does not duplicate material already published. I confirm all authors consent to publication of this Work and authorise the granting of this licence.

For peer review only

Characteristics of clinician input in Canadian funding decisions for cancer drugs: a cross-sectional, descriptive study based on CADTH reimbursement recommendations

Kristina Jenei BSN MSc[1] & Daniel E. Meyers MD MSc[2],

[1] London School of Economics and Political Science
[2] Department of Medicine, University of Calgary, Calgary, Canada
[3]

Word count (text): 2628
Tables: 3
Figure: 1

Corresponding author:
Kristina Jenei, BSN MSc
Address: Houghton Street
London, United Kingdom
WC2A 2AE Telephone: +44-7911-494-962
Email: k.jenei@lse.ac.uk

For peer review only

For peer review only - http://bmjopen.bmj.com/site/about/guidelines.xhtml

Structured Abstract

Objective: To examine characteristics of clinician input to the pan-Canadian Oncology Drug Review (pCODR) for cancer drug funding recommendations from 2016 to 2020.

Design, Setting, and Participants: Descriptive, cross-sectional study included 62 reimbursement decisions from pCODR from 2016 to 2020.

Interventions: pCODR recommendations were analyzed for the number of clinicians consulted on each submission, affiliation, number of submissions per clinician, declared financial conflict of interests (FCOIs), randomization, type of masking, primary endpoint, study phase, and whether the study demonstrated improvement in overall survival (OS) and progression free survival (PFS).

Main outcome measures: The main outcome was clinician support for the initial funding recommendation.

Results: The study consisted of 62 submissions, in which 48 included clinician input. A total of 129 unique clinicians provided 342 consultations. The majority (59%) provided input on less than five submissions, however, a small proportion (4%) consulted on over 10. Nearly all clinicians were physicians (125; 96%). From the 342 consultations, 228 declared financial conflicts (67%). The most common conflicts were payments for advisory roles (51%) and honorariums (23%). Of the 48 cancer drugs under review, clinicians recommended funding 46 (96%). Only 12 (25%) demonstrated substantial benefit, according to the ESMO-MCBS score. Drugs recommended for funding were more likely to have improved PFS and OS data. However, most cancer drugs supported by clinicians demonstrated no change in health-related quality of life (HRQoL), including one that demonstrated worsened HRQoL.

Conclusion: Clinicians offer crucial information in funding decisions. However, we found clinicians strongly supported funding nearly all cancer drugs under review, despite most not offering substantial benefit to patients nor gains in quality of life. While these drugs might be helpful options in clinical practice, funding numerous cancer drugs may be unsustainable for public health systems.

For peer review only - http://bmjopen.bmj.com/site/about/guidelines.xhtml

Strengths and Limitations

- The study examines data from CADTH reimbursement recommendations that are used to guide provincial funding decisions in Canadian provinces
- To the best of our knowledge, this study is the first to examine characteristics of clinician group input with the quality of evidence that underpin the drugs in Canadian funding decisions
- While the study uses the ESMO-MCBS tool to assess clinical value, the cohort examined is a homogenous sample of solid tumour indications which, cannot be generalised to all malignancies

For peer review only

Keywords
Health technology assessment; oncology; drug approvals; health policy; conflict of interest

For peer review only

Introduction

Country expenditure on cancer drugs has risen worldwide. Oncology medicines often account for the largest proportion of drug expenditure. Further, the costs of oncology service delivery have grown. In Europe, cancer care accounts for 30% of total hospital expenditure across Europe.[1] Indeed, sales of oncology medicines are expected to grow to $237 billion by 2024, with many new drugs under development.[2,3] In Canada, the largest proportion of Canadian public drug funding (22.9%) was spent on oncology agents.[4] Indeed, Canada is third highest for total dollars spent on pharmaceuticals among 31 countries included in The Organisation for Economic Co-operation and Development (OECD).[5] The trend of rising costs is concerning due to uncertain health gain often offered by new therapeutics. Previous research has found weak correlation between overall survival and the prices of new medicines at market launch.[6] Taken together, these challenges threaten the sustainability of publicly funded healthcare systems globally.[7–9]

Many countries with publicly funded health care systems employ health technology assessments to evaluate the value of new health technologies. These assessments often include stakeholder perspectives to ensure values perspectives are considered in the funding decision. In Canada, the Canadian Agency for Drugs and Technology in Health (CADTH) is the national health technology assessment (HTA) body that guides funding recommendations for Canadian provinces. CADTH, and its oncology assessment arm, the pan-Canadian Oncology Drug Review (pCODR)[10], evaluates medicines through a deliberative framework that includes clinical and economic evidence, clinician, and patient feedback.[11] While there are special authorisations, generally only medicines that are funded by the province are accessible to patients. Once a drug is reviewed, the pCODR Expert Review Committee (pERC)[12] — an appointed interdisciplinary body comprised of clinicians, ethicists, health economists, and patients — provide the final reimbursement recommendation. While each Canadian province independently decides whether a drug will be funded under their public drug plans, provinces often follow CADTH recommendations.[13] Since 2021, the pCODR and Common Drug Review (HTA assessment all other health interventions) has been aligned within one process. However, the responsibility of recommendations to pERC and the process for clinician group feedback remains the same.

Clinicians are involved in Canadian cancer drug funding decisions through two mechanisms. First, every drug review team includes oncologists to develop the systematic review protocol, interpret and provide real-world applicability for the study results. Once pERC reviews the clinical and economic reports and provides an initial funding recommendation, there is a second opportunity for clinician feedback. Since October 2016, CADTH provides an opportunity for additional feedback through external “calls for clinician input”. Registered clinicians, such as oncologists, pharmacists, and oncology nurses can submit feedback. While submissions can be made individually, CADTH encourages clinicians to submit in a group or health care association. Each submission must include an oncologist. This feedback is used to determine the final funding recommendation and published on the pCODR website.

For peer review only - http://bmjopen.bmj.com/site/about/guidelines.xhtml

Given trial participants are often non-representative of clinical practice, clinician and patient perspectives are important to prioritise. However, previous research exploring the role of clinicians in Canadian funding decisions found widespread financial conflict of interests (FCOIs).[14] In the United States (US), FCOIs are widespread in oncology[15] and have been found to influence prescribing patterns, formulary recommendations, research, and recommendations for clinical practice guidelines.[16] Given these associations, we sought to evaluate characteristics of clinician input beyond FCOIs, including the alignment with funding support and underlying clinical evidence for the cancer drugs under review.

For peer review only

For peer review only - http://bmjopen.bmj.com/site/about/guidelines.xhtml

Methods

We conducted a cross-sectional, descriptive study of reimbursement recommendations to pCODR for solid tumour indications between 2016 and 2020. The study examined publicly available data and therefore was exempt from institutional review board approval, in accordance with the US Department of Health and Human Services, 45 CFR §46.102(f). We adhered to the Strengthening the Reporting of Observational studies in Epidemiology (STROBE) reporting guidelines.

Source of data

A search of the pCODR website was completed to identify characteristics of physician submissions and supporting evidence. We used a sample of solid tumor indications from an existing dataset.[17] Supportive care medicines, hematologic neoplasms, pediatric indications, and biosimilars were excluded. By focusing on solid tumor indications, the dataset was homogenous which allowed us to assess the clinical benefit using the European Society for Medical Oncology Magnitude of Clinical Benefit Scale (ESMO-MCBS)[18], which was designed for solid tumour indications. We extracted the name of the drug, date and year of reimbursement recommendation and supporting clinical trial information, such as randomization, masking, overall survival and progression free survival outcomes, health related quality of life data, and estimated price (per 28-day cycle). While final drug prices are confidentially negotiated with the manufacturer after the funding recommendation, estimated prices are included in the pCODR economic evaluation reports. For more information on how these data were collected and how the ESMO-MCBS scores were calculated, see the previous publication.[17]

Clinician input on cancer drug submissions

In addition to the clinical evidence supporting each drug, we extracted data related to the clinician submissions. These data included the number of clinicians consulted on each submission, affiliation, number of submissions per clinician, and declared FCOIs. Clinicians provide feedback on the initial funding recommendation. On the submission forms, clinicians are asked whether they "agree", "disagree", or "agree in part" with the initial funding recommendation. With this feedback, pCODR can revise the final funding recommendation. While there are often numerous clinicians involved in each drug review, the feedback form is submitted collectively. When the clinicians agreed with a positive funding recommendation or disagreed with a negative funding recommendation, we coded this as "fund". When the clinicians agreed with a negative recommendation or disagreed with a positive funding recommendation, we coded this as "do not fund". When the clinician feedback was to "agree in part", we reviewed comments in the feedback documents to ensure coding was accurate.

Conflict of interest data

Despite one collective feedback form, FCOI forms are submitted individually. FCOI forms require clinicians to disclose whether they have received payments over the previous two years from any company or organization that may have direct or indirect interest in the drug under review. The forms require disclosure on the type of activity the payment was for (e.g., advisory board, conferences, honoraria, research, sponsorship

For peer review only - http://bmjopen.bmj.com/site/about/guidelines.xhtml

of events, operations, travel, among others). Clinicians must also provide the names of the manufacturers who they have received funds from, including whether these payments exceeded CAD$10,000 and whether they have personal or commercial relationships with any interest group in relation to the drug under submission. Information related to all these categories were also extracted.

Data analysis

Descriptive data and counts are presented throughout this manuscript. While FCOI documentation is submitted individually, clinician feedback is sought through groups and cannot be disaggregated further. Therefore, due to CADTH procedures, we analysed clinician group submissions. Financial conflicts of interest were analysed as individuals given available data. Data analysis was conducted using Microsoft Excel Version 16.73.

Patient and Public Involvement

No patients nor publics were involved.

For peer review only

For peer review only - http://bmjopen.bmj.com/site/about/guidelines.xhtml

Results

Table 1 reports the descriptive characteristics of clinicians that were consulted during cancer drug funding decisions. Overall, there were 62 drug funding reviews by CADTH for solid tumour indications between 2016 and 2020. Of these, clinician group input were available for 48 submissions. For 14, there were no clinician feedback published on the pCODR website (there are no reasons provided for why these submissions did not have clinician input). On average, 4 (IQR: 2-8) clinicians provided feedback per drug. In the 48 drug reviews, there was 342 submissions from clinicians for feedback on funding recommendations. However, there were only 129 unique clinicians which suggests clinicians often consulted on more than one drug. Of these, 124 (96%) were physicians and 5 (4%) were oncology pharmacists. Most clinicians (76; 59%) consulted on only one drug review. However, a small minority (4; 3%) made repeat submissions on more than 10 drugs.

An overwhelming majority of clinicians were in favour of funding the drug under review. **Table 2** reports the clinician recommendations and supporting evidence. Of the 48 submissions that included clinician input, 46 (96%) had positive clinician feedback to fund the drug. This included 12 (26%) drugs that received negative recommendations from CADTH in which clinicians submitted feedback in support of funding the drug. Only 2 (4%) submissions believed the drug should not be funded.
Of the drugs recommended for funding, most (32; 70%) did not offer substantial benefit, as per ESMO-MCBS.

In terms of clinical benefit, clinicians recommended funding 18 (39%) drugs that had data that demonstrated an OS benefit, 12 (26%) without an OS benefit, and 16 (35%) that had no data on OS. Similarly, 25 drugs (54%) that demonstrated an improved PFS benefit, 5 (11%) that did not improve PFS, and 16 drugs (35%) that did not have any data related to PFS received funding support from clinicians. Nearly two thirds of drugs (32; 70%) that received funding support by clinicians demonstrated no change in HRQoL, with one that worsened HRQoL outcomes. Most cancer drugs that received clinician funding support were between $5000 to $9,999 per month (59%), including 14 (30%) that were over $10,000 per 28-day cycle (Table 3).

We found numerous financial conflicts of interests **(Figure 1)**. Of the 48 submissions made by clinician groups, 46 had complete FCOI disclosures that were able to be analysed (9 of 342 individual documents; 3%) were incomplete. Of the 342 instances where clinicians were consulted during the drug review, 228 (67%) reported FCOIs **(Table 3)**. Of these, 149 (44%) were with the manufacturer of the drug under review. The average number of conflicts a clinician disclosed was 3 (IQR: 2 to 6). Advisory roles were the most common type of conflict (166; 51%), followed by honorariums (23%), payments to attend conferences, research, and sponsorship of events (7%, respectively). We found 9 documents (3%) that were incomplete.

For peer review only - http://bmjopen.bmj.com/site/about/guidelines.xhtml

Discussion

We sought to descriptively examine characteristics of clinicians consulted in Canadian cancer drug funding decisions from 2016 to 2020. We examined clinician recommendations and supporting evidence for the cancer drugs under review for reimbursement. Further, we explored the extent of FCOIs disclosed in clinician submissions. We found that clinicians who consult on Canadian funding decisions support funding the cancer drug under review. Of the 48 drugs, 46 (96%) received supportive funding feedback from clinicians, even when most drugs did not offer substantial benefit to patients nor improved quality of life. These findings have important implications.

First, our findings highlight a substantial proportion of new cancer medicines are introduced in the Canadian market with high prices and uncertain or negligible value. It is reasonable to assume many clinicians would prefer robust, mature evidence demonstrating improvements patient-cantered outcomes, such as improvements in overall survival and quality of life. However, in a previous study, we found that only half of cancer drugs that received a positive funding recommendation from pCODR had evidence of improved survival.[17] Similarly, the Patented Medicine Prices Review Board (PMPRB) —the federal agency responsible for regulating drug prices of new patented medicines in Canada — classified over 90% of new medicines as offering “moderate, little, or no improvement” in clinical benefit.[19] Therefore, in this ecosystem, objective and clear decision making is required. Influence from conflicts or vested interests might add strain to health budgets.

Second, our findings might highlight conflicting principal-agent relationships between national health technology assessment (HTA) bodies, pharmaceutical manufacturers, and clinicians. The duty of a clinician to care for individual patients differs from HTA bodies who attempt to maximize population health outcomes for every dollar spent. For clinicians, it might be advantageous to have numerous treatment options to offer patients, including a spectrum of available medicines given differential responses to individual therapies. However, at the national reimbursement level, funding numerous high-priced cancer drugs incurs a large budget impact. Indeed, 27 drugs (59%) that received positive funding support from clinicians were priced between $5000 to $9,999 per month. This is concerning in oncology where many high-priced cancer drugs approved through regulatory agencies often only offer marginal benefit, if any at all.[17,20,21]

Third, we found numerous conflicts of interests. Of the 342 submissions, 228 (67%) declared conflicts and nearly half (149; 44%) were directly with the manufacturer of the drug under review. While the relationship between clinician behaviour and FCOIs in Canada might warrant further investigation, we confirm previous research[14] that these findings are higher than FCOIs disclosed in US Food and Drug Administration Drug Advisory Committee meetings.[22] Previous Canadian research suggests FCOIs might influence clinician input and subsequent feedback influences the final recommendation. Lexchin found that 75% of clinicians who disagreed with a negative funding

For peer review only - http://bmjopen.bmj.com/site/about/guidelines.xhtml

recommendation declared a conflict with the manufacturer under review.[14] However, in the nine negative initial funding recommendations, pCODR changed their final recommendation once. Similarly, in our study, the initial funding recommendation was only changed once after stakeholder feedback. Further, we found 9 of 342 (3%) FCOI documents that were incomplete which raises concerns about the disclosure process. The literature about FCOIs and clinician behaviour suggests that there is a relationship between financial payments to clinicians and prescribing patterns, formulary recommendations, research, and recommendations for clinical practice guidelines.[15,16] Future research might build on our descriptive study to further examine the weight of these factors on the decision and reasons offered by clinicians in cases where positive feedback was given for drugs with negligible evidence.Our findings raise concerns about the use of stakeholder perspectives in funding decisions and are relevant to other countries outside of Canada that use similar HTA methodology. Countries, such as England, Italy, New Zealand, Australia, among others, include input from a variety of groups in funding decisions. However, Canada is the only country that includes patient and clinician group feedback in every decision. Other countries include stakeholder input as deemed necessary, such as funding a drug for a rare or severe disease. For example, the National Institute for Health and Care Excellence (England) allows stakeholders and individuals to comment on draft recommendations on a case-by-case basis. Given our findings demonstrated overwhelming support for funding nearly every drug despite evidence to suggest negligible benefit, HTA agencies might be more explicit for how stakeholder input is used in funding decisions to manage provider-induced demand.

Strengths and Limitations

Our retrospective cohort study has strengths and limitations. It is unique as it is the first evaluation of characteristics of clinician input in Canadian funding decision that includes an assessment of the supporting evidence of the drugs under review. However, we encountered limitations. First, we were limited to a cohort of cancer drugs that was accepted and reviewed by CADTH. Second, feedback forms where clinicians indicate their support for drug funding are submitted collectively, not individually. Therefore, clinician input cannot be attributed to individual views. Third, we are limited by a cohort of submissions for solid tumour indications which might differ compared to other malignancies. However, this approach allows for homogenous dataset for which clinical benefit can be measured using the ESMO-MCBS. Further, several methodological limitations have been discussed regarding the ESMO-MCBS tool[23] (use of the lower limit confidence interval, focus on primary endpoints even for studies that use unvalidated surrogate endpoints, use of QoL data, and no weighted arguments for values) which should be acknowledged given it is the measure of value within our study. Lastly, we are limited by the publicly available data in pCODR reports. Given the complexity of evaluating evidence from oncology clinical trials, these reports might not offer insight into the nuanced discussions that likely occurred throughout the pCODR deliberations.

Conclusions

For peer review only - http://bmjopen.bmj.com/site/about/guidelines.xhtml

Clinicians offer crucial information in funding decisions. However, we found overwhelming support for nearly all drugs under review, even medicines that did not demonstrate improved OS benefit nor quality of life — two of the most important patient-centered outcomes. Further, we found numerous FCOIs. These findings raise questions about the role of stakeholder input in funding decisions and possible incongruity between objectives of national health technology assessment bodies and practising clinicians.

For peer review only

Ethics approval: The study examined publicly available data and therefore was exempt from institutional review board approval, in accordance with the US Department of Health and Human Services, 45 CFR §46.102(f).

Contributor statement: Ms. Jenei lead the analysis, drafting, and dissemination. Dr. Meyers were involved in the drafting and editing of the manuscript.

Competing interests: Ms. Jenei and Dr. Meyers report no conflicts.

Funding statement: Ms. Jenei is supported by a Canadian Institutes of Health Research Doctoral Foreign Study Award (181603). The funder was not involved in the manuscript.

Data sharing statement: Data are available upon reasonable request.

For peer review only

References

1. Simoens S, KU Leuven, Leuven, Belgium, Van Harten W, et al. What Happens when the Cost of Cancer Care Becomes Unsustainable? *European Oncology & Haematology*. 2017;13(02):108. doi:10.17925/EOH.2017.13.02.108

2. IMSH Institute Global Oncology Trend 2015 2020 Report | PDF | Non Small Cell Lung Carcinoma | Pharmaceutical Drug. Scribd. Accessed May 24, 2023. https://www.scribd.com/document/323179495/IMSH-Institute-Global-Oncology-Trend-2015-2020-Report

3. Evaluate Pharma. *World Preview 2019, Outlook to 2024*. Accessed May 24, 2023. https://info.evaluate.com/rs/607-YGS-364/images/EvaluatePharma_World_Preview_2019.pdf

4. Canadian Institute for Health Information. *Prescribed Drug Spending in Canada*. Canadian Institute for Health Information; 2020.

5. OECD. Pharmaceutical spending. Published 2023. Accessed December 1, 2021. https://data.oecd.org/healthres/pharmaceutical-spending.htm#indicator-chart

6. Jenei K, Meyers D, Gyawali B. Assessment of Price and Clinical Benefit of Cancer Drugs in Canada, 2011-2020. *JAMA Network Open*. 2023;6(1):e2253438. doi:10.1001/jamanetworkopen.2022.53438

7. Cohen D. Cancer drugs: high price, uncertain value. *BMJ (Clinical research ed)*. 2017;359:j4543. doi:https://dx.doi.org/10.1136/bmj.j4543

8. Haycox A. Why Cancer? *Pharmacoeconomics*. 2016;34:625-627. doi:10.1007/s40273-016-0413-0

9. Godman B, Hill A, Simoens S, et al. Potential approaches for the pricing of cancer medicines across Europe to enhance the sustainability of healthcare systems and the implications. *Expert Rev Pharmacoecon Outcomes Res*. 2021;21(4):527-540. doi:10.1080/14737167.2021.1884546

10. About the pan-Canadian Oncology Drug Review (pCODR) | CADTH.ca. 2019. Accessed August 20, 2019. https://cadth.ca/pcodr/about-pcodr

11. CADTH. About CADTH. 2019. Accessed July 17, 2019. https://www.cadth.ca/about-cadth

12. Health CA for D and T in. *PCODR Expert Review Committee Deliberative Framework*.; 2016:1-7. Accessed October 30, 2019. www.cadth.ca/pcodr

13. Gamble JM, Weir DL, Johnson JA, Eurich DT. Analysis of drug coverage before and after the implementation of Canada's Common Drug Review. *CMAJ*. 2011;183(17):E1259. doi:10.1503/CMAJ.110670/-/DC1

For peer review only - http://bmjopen.bmj.com/site/about/guidelines.xhtml

14. Lexchin J. Financial conflicts of interest of clinicians making submissions to the pan-Canadian Oncology Drug Review: a descriptive study. *BMJ Open*. 2019;9:30750. doi:10.1136/bmjopen-2019-030750

15. Meyers DE, Chisamore TM, McInnes MDF, Gyawali B, Prasad V, Booth CM. Industry payments to US physicians for cancer therapeutics: An analysis of the 2016–2018 open payments datasets. *Journal of Cancer Policy*. 2021;28:100283. doi:10.1016/J.JCPO.2021.100283

16. Mitchell AP, Trivedi NU, Gennarelli RL, et al. Are Financial Payments from the Pharmaceutical Industry Associated with Physician Prescribing? A Systematic Review. *Annals of Internal Medicine*. 2021;174(3):353-361. doi:10.7326/M20-5665

17. Meyers DE, Jenei K, Chisamore TM, Gyawali B. Evaluation of the Clinical Benefit of Cancer Drugs Submitted for Reimbursement Recommendation Decisions in Canada. *JAMA Internal Medicine*. 2021;181(4):499-508. doi:10.1001/jamainternmed.2020.8588

18. Cherny NI, Dafni U, Bogaerts J, et al. ESMO-Magnitude of Clinical Benefit Scale version 1.1. *Annals of Oncology*. 2017;28(10):2340-2366. doi:10.1093/ANNONC/MDX310

19. Lexchin J. Canadian status of “drugs to avoid” in 2017: a descriptive analysis. *CMAJ Open*. 2018;6(3):E430. doi:10.9778/CMAJO.20180049

20. Fojo T, Mailankody S, Lo A. Unintended consequences of expensive cancer therapeutics - The pursuit of marginal indications and a me-too mentality that stifles innovation and creativity: The John Conley lecture. *JAMA Otolaryngology - Head and Neck Surgery*. 2014;140(12):1225-1236. doi:10.1001/jamaoto.2014.1570

21. Cherla A, Naci H, Kesselheim AS, Gyawali B, Mossialos E. Assessment of Coverage in England of Cancer Drugs Qualifying for US Food and Drug Administration Accelerated Approval. *JAMA Internal Medicine*. 2021;181(4):490-498. doi:10.1001/JAMAINTERNMED.2020.8441

22. Lurie P, Almeida CM, Stine N, Stine AR, Wolfe SM. Financial Conflict of Interest Disclosure and Voting Patterns at Food and Drug Administration Drug Advisory Committee Meetings. *JAMA*. 2006;295(16):1921-1928. doi:10.1001/JAMA.295.16.1921

23. Wild C, Grössmann N, Bonanno PV, et al. Utilisation of the ESMO-MCBS in practice of HTA. *Ann Oncol*. 2016;27(11):2134-2136. doi:10.1093/annonc/mdw297

For peer review only - http://bmjopen.bmj.com/site/about/guidelines.xhtml

Table 1. Characteristics of clinicians that submitted input to the pan-Canadian Oncology Drug Review

**Characteristics of individual clinicians consulted**	
Total number of clinician feedback submissions	342 (100)
Unique clinicians	129 (100)
Number of funding decisions consulted	
One	76 (59)
Two	16 (12)
3 to 10	33 (26)
More than 10	4 (3)
Affiliation	
Physician	124 (96)
Oncology pharmacist	5 (4)
**Characteristic of clinician group submissions**	
Reimbursement decisions (2016-2020)	62 (100)
Reimbursement decisions with clinician submissions[a]	48 (100)
Clinician recommendation	
Fund	46 (96)
Do not fund	2 (4)
Median number of clinicians that offered feedback per drug review (IQR)	4 (2-8)

[a]Study sample

For peer review only

Table 2. Clinician group funding recommendation and supporting evidence

	Clinician group recommendation (n=48)	
	Fund (n=46)	Do not fund (n=2)
CADTH decision		
Positive	29 (63)	0 (0)
Negative	12 (26)	2 (100)
Submission w/conflicts		
Yes	44 (96)	1 (50)
No	2 (4)	1 (50)
Submissions w/ conflicts with manufacturer		
Yes	34 (74)	1 (50)
No	12 (26)	1 (50)
ESMO-MCBS Score		
Substantial benefit	14 (30)	2 (100)
No substantial benefit	32 (70)	0 (0)
RCT		
Yes	34 (74)	2 (100)
No	12 (26)	0 (0)
Phase		
1	3 (7)	0 (0)
2	10 (22)	0 (0)
3	33 (72)	2 (100)
Masked studies		
Yes	20 (43)	2 (100)
No	14 (30)	0 (0)
NR	12 (26)	0 (0)
OS		
Improved	18 (39)	0 (0)
Not improved	12 (26)	1 (50)
NA	16 (35)	1 (50)
PFS Data		
Improved	25 (54)	0 (0)
Not improved	5 (11)	0 (0)
NA	16 (35)	2 (100)
HRQoL		
Improved	5 (11)	0 (0)
No change	32 (70)	2 (100)
Worsen	1 (2)	0 (0)
NA	5 (11)	0 (0)
Price (per 28 cycle)		
Under $5000	5 (11)	0 (0)
$5000 to $9,999	27 (59)	1 (50)
Over $10,000	14 (30)	1 (50)

For peer review only

Table 3. Financial conflict of interests among clinicians consulted on drug funding decisions

Characteristic	Submissions (n=342)
Clinician submissions declaring conflicts	228 (67)
Clinician submissions with conflicts with the manufacturer of the drug under review	149 (44)
Avg number of conflicts per submission	3 (2-6)
Types of conflicts reported	
Advisory role	166 (51)
Honoraria	74 (23)
Conference	22 (7)
Research	22 (7)
Events	23 (7)
Operations	2 (1)
Travel	3 (1)
Speaking	12 (1)
Incomplete COI documentation	9 (3)

For peer review only

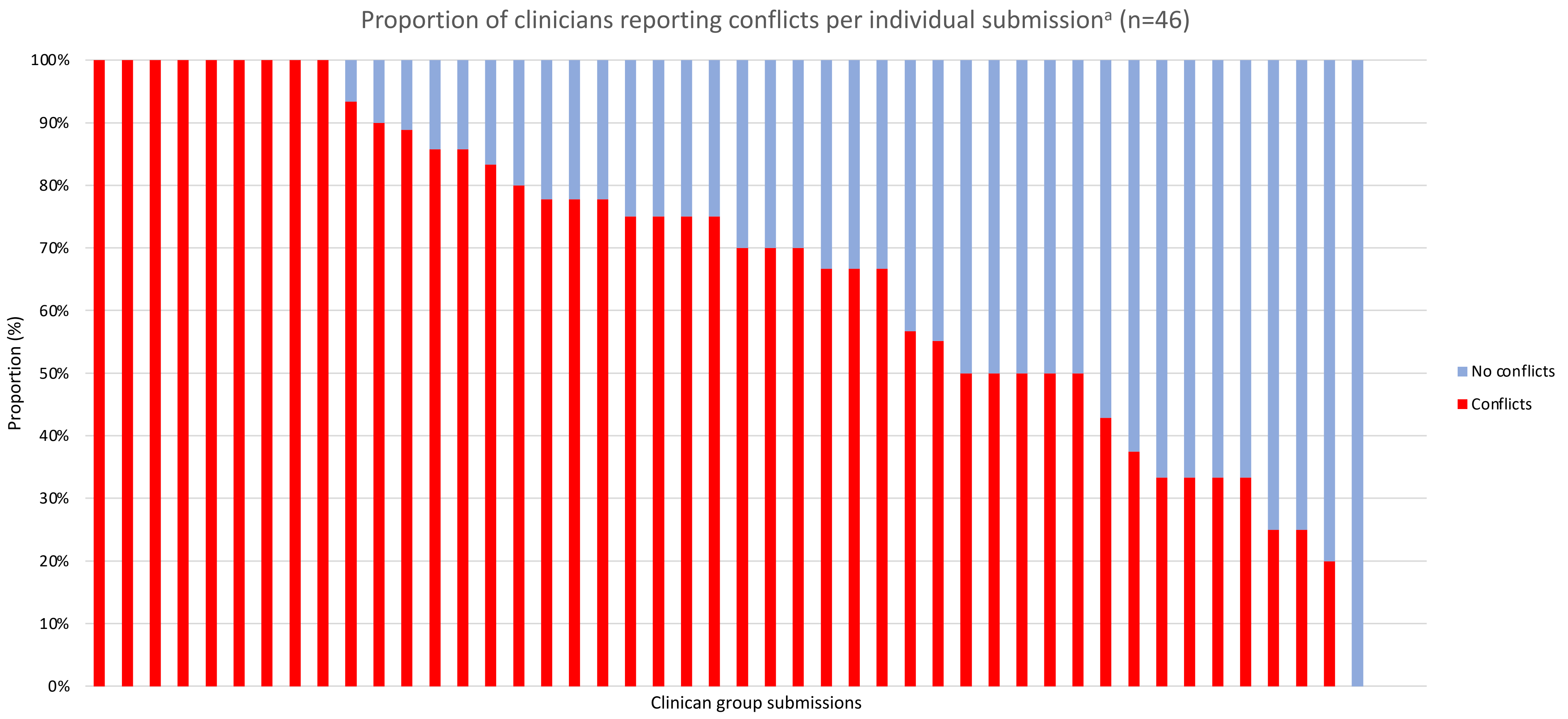

[a]Two submissions were removed from the figure due to incomplete information

STROBE Statement—Checklist of items that should be included in reports of ***cohort studies***

Characteristics of clinician input in Canadian funding decisions for cancer drugs

	Item No	Recommendation	Page No
**Title and abstract**	1	(*a*) Indicate the study's design with a commonly used term in the title or the abstract<br>(*b*) Provide in the abstract an informative and balanced summary of what was done and what was found	1-2
**Introduction**			
Background/rationale	2	Explain the scientific background and rationale for the investigation being reported	5
Objectives	3	State specific objectives, including any prespecified hypotheses	5
**Methods**			
Study design	4	Present key elements of study design early in the paper	6
Setting	5	Describe the setting, locations, and relevant dates, including periods of recruitment, exposure, follow-up, and data collection	6
Participants	6	(*a*) Give the eligibility criteria, and the sources and methods of selection of participants. Describe methods of follow-up<br>(*b*) For matched studies, give matching criteria and number of exposed and unexposed	N/A
Variables	7	Clearly define all outcomes, exposures, predictors, potential confounders, and effect modifiers. Give diagnostic criteria, if applicable	6
Data sources/ measurement	8*	For each variable of interest, give sources of data and details of methods of assessment (measurement). Describe comparability of assessment methods if there is more than one group	6
Bias	9	Describe any efforts to address potential sources of bias	NA
Study size	10	Explain how the study size was arrived at	6
Quantitative variables	11	Explain how quantitative variables were handled in the analyses. If applicable, describe which groupings were chosen and why	6
Statistical methods	12	(*a*) Describe all statistical methods, including those used to control for confounding<br>(*b*) Describe any methods used to examine subgroups and interactions<br>(*c*) Explain how missing data were addressed<br>(*d*) If applicable, explain how loss to follow-up was addressed<br>(*e*) Describe any sensitivity analyses	NA (descriptive Page 6)
**Results**			
Participants	13*	(a) Report numbers of individuals at each stage of study—eg numbers potentially eligible, examined for eligibility, confirmed eligible, included in the study, completing follow-up, and analysed<br>(b) Give reasons for non-participation at each stage<br>(c) Consider use of a flow diagram	8
Descriptive data	14*	(a) Give characteristics of study participants (eg demographic, clinical, social) and information on exposures and potential confounders<br>(b) Indicate number of participants with missing data for each variable of interest	8

For peer review only - http://bmjopen.bmj.com/site/about/guidelines.xhtml

		(c) Summarise follow-up time (eg, average and total amount)	
Outcome data	15*	Report numbers of outcome events or summary measures over time	8

For peer review only

For peer review only - http://bmjopen.bmj.com/site/about/guidelines.xhtml

Main results	16	(*a*) Give unadjusted estimates and, if applicable, confounder-adjusted estimates and their precision (eg, 95% confidence interval). Make clear which confounders were adjusted for and why they were included<br>(*b*) Report category boundaries when continuous variables were categorized<br>(*c*) If relevant, consider translating estimates of relative risk into absolute risk for a meaningful time period	8
Other analyses	17	Report other analyses done—eg analyses of subgroups and interactions, and sensitivity analyses	NA
**Discussion**			
Key results	18	Summarise key results with reference to study objectives	
Limitations	19	Discuss limitations of the study, taking into account sources of potential bias or imprecision. Discuss both direction and magnitude of any potential bias	9
Interpretation	20	Give a cautious overall interpretation of results considering objectives, limitations, multiplicity of analyses, results from similar studies, and other relevant evidence	9
Generalisability	21	Discuss the generalisability (external validity) of the study results	9/10
**Other information**			
Funding	22	Give the source of funding and the role of the funders for the present study and, if applicable, for the original study on which the present article is based	1

*Give information separately for exposed and unexposed groups.

Note: An Explanation and Elaboration article discusses each checklist item and gives methodological background and published examples of transparent reporting. The STROBE checklist is best used in conjunction with this article (freely available on the Web sites of PLoS Medicine at http://www.plosmedicine.org/, Annals of Internal Medicine at http://www.annals.org/, and Epidemiology at http://www.epidem.com/). Information on the STROBE Initiative is available at http://www.strobe-statement.org.

For peer review only

BMJ Open

Characteristics of clinician input in Canadian funding decisions for cancer drugs: a cross-sectional study based on CADTH reimbursement recommendations

Journal:	*BMJ Open*
Manuscript ID	bmjopen-2022-066378.R2
Article Type:	Original research
Date Submitted by the Author:	07-Aug-2023
Complete List of Authors:	Jenei, Kristina ; The London School of Economics and Political Science, Department of Health Policy<br>Meyers, Daniel E.; University of Calgary, Department of Medicine
**Primary Subject Heading**:	Health economics
Secondary Subject Heading:	Oncology, Evidence based practice
Keywords:	Health policy < HEALTH SERVICES ADMINISTRATION & MANAGEMENT, HEALTH ECONOMICS, ONCOLOGY

SCHOLARONE™
Manuscripts

I, the Submitting Author has the right to grant and does grant on behalf of all authors of the Work (as defined in the below author licence), an exclusive licence and/or a non-exclusive licence for contributions from authors who are: i) UK Crown employees; ii) where BMJ has agreed a CC-BY licence shall apply, and/or iii) in accordance with the terms applicable for US Federal Government officers or employees acting as part of their official duties; on a worldwide, perpetual, irrevocable, royalty-free basis to BMJ Publishing Group Ltd ("BMJ") its licensees and where the relevant Journal is co-owned by BMJ to the co-owners of the Journal, to publish the Work in this journal and any other BMJ products and to exploit all rights, as set out in our licence.

The Submitting Author accepts and understands that any supply made under these terms is made by BMJ to the Submitting Author unless you are acting as an employee on behalf of your employer or a postgraduate student of an affiliated institution which is paying any applicable article publishing charge ("APC") for Open Access articles. Where the Submitting Author wishes to make the Work available on an Open Access basis (and intends to pay the relevant APC), the terms of reuse of such Open Access shall be governed by a Creative Commons licence – details of these licences and which Creative Commons licence will apply to this Work are set out in our licence referred to above.

Other than as permitted in any relevant BMJ Author's Self Archiving Policies, I confirm this Work has not been accepted for publication elsewhere, is not being considered for publication elsewhere and does not duplicate material already published. I confirm all authors consent to publication of this Work and authorise the granting of this licence.

For peer review only

Characteristics of clinician input in Canadian funding decisions for cancer drugs: a cross-sectional study based on CADTH reimbursement recommendations

Kristina Jenei BSN MSc[1] & Daniel E. Meyers MD MSc[2],

[1] London School of Economics and Political Science
[2] Department of Medicine, University of Calgary, Calgary, Canada

Word count (text): 2990
Tables: 5
Figure: 1

Corresponding author:
Kristina Jenei, BSN MSc
Address: Houghton Street
London, United Kingdom
WC2A 2AE Telephone: +44-7911-494-962
Email: k.jenei@lse.ac.uk

For peer review only - http://bmjopen.bmj.com/site/about/guidelines.xhtml

Structured Abstract

Objective: To examine characteristics of clinician input to the pan-Canadian Oncology Drug Review (pCODR) for cancer drug funding recommendations from 2016 to 2020.

Design, Setting, and Participants: Descriptive, cross-sectional study included 62 reimbursement decisions from pCODR from 2016 to 2020.

Interventions: pCODR recommendations were analyzed for the number of clinicians consulted on each submission, affiliation, number of submissions per clinician, declared financial conflict of interests (FCOIs), randomization, type of masking, primary endpoint, study phase, and whether the study demonstrated improvement in overall survival (OS) and progression free survival (PFS).

Main outcome measures: The main outcome was clinician support for the initial funding recommendation. Secondary outcome measure was the association between clinician FCOI and clinical benefit in positive recommendations.

Results: The study consisted of 62 submissions, in which 48 included clinician input. A total of 129 unique clinicians provided 342 consultations. The majority (59%) provided input on less than five submissions, however, a small proportion (4%) consulted on over 10. Nearly all clinicians were physicians (125; 96%). From the 342 consultations, 228 declared financial conflicts (67%). The most common conflicts were payments for advisory roles (51%) and honorariums (23%). Of the 48 cancer drugs under review, clinicians recommended funding 46 (96%). Only 12 (25%) demonstrated substantial benefit, according to the ESMO-MCBS score. Drugs recommended for funding were more likely to have improved PFS and OS data. However, most cancer drugs supported by clinicians demonstrated no change in health-related quality of life (HRQoL), including one that demonstrated worsened HRQoL. There was no statistically significant difference between FCOI status and recommending drugs with health gains.

Conclusion: Clinicians offer crucial information in funding decisions. However, we found clinicians strongly supported funding nearly all cancer drugs under review, despite most not offering substantial benefit to patients nor gains in quality of life. While these drugs might be helpful options in clinical practice, funding numerous cancer drugs may be unsustainable for public health systems.

For peer review only - http://bmjopen.bmj.com/site/about/guidelines.xhtml

Strengths and Limitations

- The study examines data from CADTH reimbursement recommendations that are used to guide provincial funding decisions in Canadian provinces
- To the best of our knowledge, this study is the first to examine characteristics of clinician group input with the quality of evidence that underpin the drugs in Canadian funding decisions
- While the study uses the ESMO-MCBS tool to assess clinical value, the cohort examined is a homogenous sample of solid tumour indications which, cannot be generalised to all malignancies

For peer review only

Keywords
Health technology assessment; oncology; drug approvals; health policy; conflict of interest

For peer review only

Introduction

Country expenditure on cancer drugs has risen worldwide. Oncology medicines often account for the largest proportion of drug expenditure. Further, the costs of oncology service delivery have grown. In Europe, cancer care accounts for 30% of total hospital expenditure across Europe.(1) Indeed, sales of oncology medicines are expected to grow to $237 billion by 2024, with many new drugs under development.(2,3) In Canada, the largest proportion of Canadian public drug funding (22.9%) was spent on oncology agents.(4) Indeed, Canada is third highest for total dollars spent on pharmaceuticals among 31 countries included in The Organisation for Economic Co-operation and Development (OECD).(5) The trend of rising costs is concerning due to uncertain or negligible health gain often offered by new therapeutics.(6) Furthermore, previous research has found weak correlation between overall survival and the prices of new medicines at market launch.(7) Taken together, these challenges threaten the sustainability of publicly funded healthcare systems globally.(8–10)

Many countries with publicly funded health care systems employ health technology assessments to evaluate the value of new health technologies. These assessments often include stakeholder perspectives to ensure values perspectives are considered in the funding decision. In Canada, the Canadian Agency for Drugs and Technology in Health (CADTH) is the national health technology assessment (HTA) body that guides funding recommendations for Canadian provinces. CADTH, and its oncology assessment arm, the pan-Canadian Oncology Drug Review (pCODR)(11), evaluates medicines through a deliberative framework that includes clinical and economic evidence, clinician, and patient feedback.(12) While there are special authorisations, generally only medicines that are funded by the province are accessible to patients. Once a drug is reviewed, the pCODR Expert Review Committee (pERC)(13) — an appointed interdisciplinary body comprised of clinicians, ethicists, health economists, and patients — provide the final reimbursement recommendation. While each Canadian province independently decides whether a drug will be funded under their public drug plans, provinces often follow CADTH recommendations.(14) Since 2021, the pCODR and Common Drug Review (HTA assessment all other health interventions) has been aligned within one process. However, the responsibility of recommendations to pERC and the process for clinician group feedback remains the same.

Clinicians are involved in Canadian cancer drug funding decisions through two mechanisms. First, every drug review team includes oncologists to develop the systematic review protocol, interpret and provide real-world applicability for the study results. Once pERC reviews the clinical and economic reports and provides an initial funding recommendation, there is a second opportunity for clinician feedback. Since October 2016, CADTH provides an opportunity for additional feedback through external “calls for clinician input”. Registered clinicians, such as oncologists, pharmacists, and oncology nurses can submit feedback. While submissions can be made individually, CADTH encourages clinicians to submit in a group or health care association. Each submission must include an oncologist. This feedback is used to determine the final funding recommendation and published on the pCODR website.

For peer review only - http://bmjopen.bmj.com/site/about/guidelines.xhtml

Given trial participants are often non-representative of clinical practice, clinician and patient perspectives are important to prioritise to understand the value of medicine in real world settings. However, previous research exploring the role of clinicians in Canadian funding decisions found widespread financial conflict of interests (FCOIs).(15) In the United States (US), FCOIs are widespread in oncology(16) and have been found to influence prescribing patterns, formulary recommendations, research, and recommendations for clinical practice guidelines.(17) Given these associations, we sought to evaluate characteristics of clinician input beyond FCOIs, including the alignment between funding support and underlying clinical evidence for the cancer drugs under review.

For peer review only

For peer review only - http://bmjopen.bmj.com/site/about/guidelines.xhtml

Methods

We conducted a cross-sectional, descriptive study of reimbursement recommendations to pCODR for solid tumour indications between 2016 and 2020. The study examined publicly available data and therefore was exempt from institutional review board approval, in accordance with the US Department of Health and Human Services, 45 CFR §46.102(f). We adhered to the Strengthening the Reporting of Observational studies in Epidemiology (STROBE) reporting guidelines.

Source of data

A search of the pCODR website was completed to identify characteristics of physician submissions and supporting evidence. We used a sample of solid tumor indications from an existing dataset.(6) Supportive care medicines, hematologic neoplasms, pediatric indications, and biosimilars were excluded. By focusing on solid tumor indications, the dataset was homogenous which allowed us to assess the clinical benefit using the European Society for Medical Oncology Magnitude of Clinical Benefit Scale (ESMO-MCBS)(18), which was designed for solid tumour indications. We extracted the name of the drug, date and year of reimbursement recommendation and supporting clinical trial information, such as randomization, masking, overall survival and progression free survival outcomes, health related quality of life data, and estimated price (per 28-day cycle). While final drug prices are confidentially negotiated with the manufacturer after the funding recommendation, estimated prices are included in the pCODR economic evaluation reports. For more information on how these data were collected and how the ESMO-MCBS scores were calculated, see the previous publication.(6)

Clinician input on cancer drug submissions

In addition to the clinical evidence supporting each drug, we extracted data related to the clinician submissions. These data included the number of clinicians consulted on each submission, affiliation, number of submissions per clinician, and declared FCOIs. Clinicians provide feedback on the initial funding recommendation. On the submission forms, clinicians are asked whether they “agree”, “disagree”, or “agree in part” with the initial funding recommendation. With this feedback, pCODR can revise the final funding recommendation. While there are often numerous clinicians involved in each drug review, the feedback form is submitted collectively. When the clinicians agreed with a positive funding recommendation or disagreed with a negative funding recommendation, we coded this as “fund”. When the clinicians agreed with a negative recommendation or disagreed with a positive funding recommendation, we coded this as “do not fund”. When the clinician feedback was to “agree in part”, we reviewed comments in the feedback documents to ensure coding was accurate.

Conflict of interest data

Despite one collective feedback form, FCOI forms are submitted individually. FCOI forms require clinicians to disclose whether they have received payments over the previous two years from any company or organization that may have direct or indirect interest in the drug under review. The forms require disclosure on the type of activity the payment was for (e.g., advisory board, conferences, honoraria, research, sponsorship

For peer review only - http://bmjopen.bmj.com/site/about/guidelines.xhtml

of events, operations, travel, among others). Clinicians must also provide the names of the manufacturers who they have received funds from, including whether these payments exceeded CAD$10,000 and whether they have personal or commercial relationships with any interest group in relation to the drug under submission. Information related to all these categories were also extracted.

Data analysis

Descriptive data and counts are presented throughout this manuscript. While FCOI documentation is submitted individually, clinician feedback is sought through groups and cannot be disaggregated further. Therefore, due to CADTH procedures, we analysed clinician group submissions. Financial conflicts of interest were analysed as individuals given available data. We explored the association of FCOI status (conflict with the company, conflict with another company, no conflict) in recommending drugs with meaningful benefit (defined as improvements in ESMO-MCBS score, OS benefit, and HRQoL status). The purpose of this analysis was to understand the role of FCOIs in recommending drugs with or without health gains. Furthermore, we examine the association between overall funding recommendation (fund/do not fund) with FCOI status. Both analyses used Fisher's exact test. Data analysis was conducted using Microsoft Excel (version 16.73) and R Statistical Software (version 2023.06.1+524).

Patient and Public Involvement

No patients nor publics were involved.

For peer review only - http://bmjopen.bmj.com/site/about/guidelines.xhtml

Results

Table 1 reports the descriptive characteristics of clinicians that were consulted during cancer drug funding decisions. Overall, there were 62 drug funding reviews by CADTH for solid tumour indications between 2016 and 2020. Of these, clinician group input were available for 48 submissions. For 14, there were no clinician feedback published on the pCODR website (there are no reasons provided for why these submissions did not have clinician input). On average, 4 (IQR: 2-8) clinicians provided feedback per drug. In the 48 drug reviews, there was 342 submissions from clinicians for feedback on funding recommendations. However, there were only 129 unique clinicians which suggests clinicians often consulted on more than one drug. Of these, 124 (96%) were physicians and 5 (4%) were oncology pharmacists. Most clinicians (76; 59%) consulted on only one drug review. However, a small minority (4; 3%) made repeat submissions on more than 10 drugs.

An overwhelming majority of clinicians were in favour of funding the drug under review. **Table 2** reports the clinician recommendations and supporting evidence. Of the 48 submissions that included clinician input, 46 (96%) had positive clinician feedback to fund the drug. This included 12 (26%) drugs that received negative recommendations from CADTH in which clinicians submitted feedback in support of funding the drug. Only 2 (4%) submissions believed the drug should not be funded.
Of the drugs recommended for funding, most (32; 70%) did not offer substantial benefit, as per ESMO-MCBS.

In terms of clinical benefit, clinicians recommended funding 18 (39%) drugs that had data that demonstrated an OS benefit, 12 (26%) without an OS benefit, and 16 (35%) that had no data on OS. Similarly, 25 drugs (54%) that demonstrated an improved PFS benefit, 5 (11%) that did not improve PFS, and 16 drugs (35%) that did not have any data related to PFS received funding support from clinicians. Nearly two thirds of drugs (32; 70%) that received funding support by clinicians demonstrated no change in HRQoL, with one that worsened HRQoL outcomes. Most cancer drugs that received clinician funding support were between $5000 to $9,999 per month (59%), including 14 (30%) that were over $10,000 per 28-day cycle (Table 3).

We found numerous financial conflicts of interests **(Figure 1)**. Of the 48 submissions made by clinician groups, 46 had complete FCOI disclosures that were able to be analysed (9 of 342 individual documents; 3%) were incomplete. Of the 342 instances where clinicians were consulted during the drug review, 228 (67%) reported FCOIs **(Table 3)**. Of these, 149 (44%) were with the manufacturer of the drug under review. The average number of conflicts a clinician disclosed was 3 (IQR: 2 to 6). Advisory roles were the most common type of conflict (166; 51%), followed by honorariums (23%), payments to attend conferences, research, and sponsorship of events (7%, respectively). We found 9 documents (3%) that were incomplete.

Overall, we found clinicians who had conflicts (either the manufacturer or with another company) were more likely to want to fund the drug ($p<0.001$). We extended this analysis to understand the role of FCOIs in recommending drugs with or without

For peer review only - http://bmjopen.bmj.com/site/about/guidelines.xhtml

meaningful clinical benefit in positive clinician group recommendations (**Table).** We found that there was no statistically significant difference between clinician FCOI status and clinical benefit per ESMO-MCBS score, overall survival benefit, nor improvements with health-related quality of life.

For peer review only

Discussion

We sought to examine characteristics of clinicians consulted in Canadian cancer drug funding decisions from 2016 to 2020. We examined clinician recommendations and supporting evidence for the cancer drugs under review for reimbursement. Further, we explored the extent of FCOIs disclosed in clinician submissions and their association with recommending drugs with meaningful clinical benefit. We found that clinicians who consult on Canadian funding decisions support funding the cancer drug under review. Of the 48 drugs, 46 (96%) received supportive funding feedback from clinicians, even when most drugs did not offer substantial benefit to patients nor improved quality of life. These findings have important implications.

First, our findings highlight a substantial proportion of new cancer medicines are introduced in the Canadian market with high prices and uncertain or negligible value. It is reasonable to assume many clinicians would prefer robust, mature evidence demonstrating improvements patient-cantered outcomes, such as improvements in overall survival and quality of life. However, in a previous study, we found that only half of cancer drugs that received a positive funding recommendation from pCODR had evidence of improved survival.(6) Similarly, the Patented Medicine Prices Review Board (PMPRB) —the federal agency responsible for regulating drug prices of new patented medicines in Canada — classified over 90% of new medicines as offering "moderate, little, or no improvement" in clinical benefit.(19) Therefore, in this ecosystem, objective and clear decision making is required. Influence from conflicts or vested interests might add strain to health budgets.

Second, our findings might highlight conflicting principal-agent relationships between national health technology assessment (HTA) bodies, pharmaceutical manufacturers, and clinicians. The duty of a clinician to care for individual patients differs from HTA bodies who attempt to maximize population health outcomes for every dollar spent. For clinicians, it might be advantageous to have numerous treatment options to offer patients, including a spectrum of available medicines given differential responses to individual therapies. However, at the national reimbursement level, funding numerous high-priced cancer drugs incurs a large budget impact. Indeed, 27 drugs (59%) that received positive funding support from clinicians were priced between $5000 to $9,999 per month. This is concerning in oncology where many high-priced cancer drugs approved through regulatory agencies often only offer marginal benefit, if any at all.(6,20,21)

Third, we found numerous conflicts of interests. Of the 342 submissions, 228 (67%) clinicians declared conflicts and nearly half were directly with the manufacturer of the drug under review. Clinicians that declared FCOIs with the manufacturer of the submission were more likely to recommend funding the drug. However, there was no statistically significant differences between the distribution of individual clinician FCOIs and recommendation for drugs with meaningful clinical benefit. This is an interesting finding that warrants further investigation to understand whether a true cause exists but may be related to the previously discussed desire to have numerous options for patients

For peer review only - http://bmjopen.bmj.com/site/about/guidelines.xhtml

given treatment responses that may differ from the clinical trial results. Given the overwhelming support for nearly all drugs in our sample, this finding may be skewed. Future research might repeat this study with the inclusion of haematological malignancies given the recently released ESMO-MCBS for this population.(22) Our results are aligned with previous Canadian research. Lexchin found that 75% of clinicians who disagreed with a negative funding recommendation declared a conflict with the manufacturer under review.(15) However, in the nine negative initial funding recommendations, pCODR changed their final recommendation once. Similarly, in our study, the initial funding recommendation was only changed once after stakeholder feedback. Further, we found 9 of 342 (3%) FCOI documents that were incomplete which raises concerns about the disclosure process. The literature about FCOIs and clinician behaviour suggests that there is a relationship between financial payments to clinicians and prescribing patterns, formulary recommendations, research, and recommendations for clinical practice guidelines.(16,17)

Our findings raise concerns about the use of stakeholder perspectives in funding decisions and are relevant to other countries outside of Canada that use similar HTA methodology. Countries, such as England, Italy, New Zealand, Australia, among others, include input from a variety of external groups in funding decisions.(23) However, Canada is the only country that includes patient and clinician group feedback in every decision. Other countries include stakeholder input as deemed necessary, such as funding a drug for a rare or severe disease. While CADTH includes patient and clinician group feedback to ensure fair decisions, the funding recommendations from these groups are complicated by numerous FCOIs and overwhelmingly positive support for the drug. Furthermore, we found several clinicians that consult on decisions more than once, with a small proportion consulting on over 10. Repeat consultations may be warranted by the nature of oncology, where novel therapeutics treat small, specialized populations, similar to those of rare diseases. To manage provider-induced demand, CADTH may want to reconsider how the agency engages with clinicians, or whether the agency ought to incorporate external stakeholder feedback on every funding decision, given overwhelming support for funding nearly every drug independent of clinical benefit. Furthermore, CADTH, among other HTA bodies, may want to be more explicit for how stakeholder input is used in funding decisions.

Strengths and Limitations

Our retrospective cohort study has strengths and limitations. It is unique as it is the first evaluation of characteristics of clinician input in Canadian funding decision that includes an assessment of the supporting evidence of the drugs under review. However, we encountered limitations. First, we were limited to a cohort of cancer drugs that was accepted and reviewed by CADTH. Second, feedback forms where clinicians indicate their support for drug funding are submitted collectively, not individually. Therefore, clinician input cannot be attributed to individual views. However, we were able to examine FCOI status (submitted individually) and the clinical benefit status of the recommended drug. Third, we are limited by a cohort of submissions for solid tumour indications which might differ compared to other malignancies. However, this approach allows for homogenous dataset for which clinical benefit can be measured using the

For peer review only

ESMO-MCBS. Further, several methodological limitations have been discussed regarding the ESMO-MCBS tool(24) (use of the lower limit confidence interval, focus on primary endpoints even for studies that use unvalidated surrogate endpoints, use of QoL data, and no weighted arguments for values) which should be acknowledged given it is the measure of value within our study. Lastly, we are limited by the publicly available data in pCODR reports. Given the complexity of evaluating evidence from oncology clinical trials, these reports might not offer insight into the nuanced discussions that likely occurred throughout the pCODR deliberations. Future research might build on our study to further examine the effects of FCOIs on the CADTH decision with more sophisticated statistical methods or qualitative analysis.

Conclusions

Clinicians offer crucial information in funding decisions. However, we found overwhelming support for nearly all drugs under review, even for medicines that did not demonstrate improved OS benefit nor quality of life — two of the most important patients-centred outcomes. Further, we found numerous FCOIs. These findings raise questions about the role of stakeholder input in HTA funding decisions and possible incongruity between objectives of national health technology assessment bodies and practising clinicians.

For peer review only

For peer review only - http://bmjopen.bmj.com/site/about/guidelines.xhtml

Ethics approval: The study examined publicly available data and therefore was exempt from institutional review board approval.

Contributor statement: Ms. Jenei lead the analysis, drafting, and dissemination. Dr. Meyers were involved in the drafting and editing of the manuscript.

Competing interests: Ms. Jenei and Dr. Meyers report no conflicts.

Funding statement: Ms. Jenei is supported by a Canadian Institutes of Health Research Doctoral Foreign Study Award (181603). The funder was not involved in the manuscript.

Data sharing statement: Data are available upon reasonable request.

For peer review only

References

1. Simoens S, KU Leuven, Leuven, Belgium, Van Harten W, University Twente, Enschede, The Netherlands, Rijnstate Hospital, Arnhem, The Netherlands, Lopes G, et al. What Happens when the Cost of Cancer Care Becomes Unsustainable? European Oncology & Haematology. 2017;13(02):108.

2. Scribd [Internet]. [cited 2023 May 24]. IMSH Institute Global Oncology Trend 2015 2020 Report | PDF | Non Small Cell Lung Carcinoma | Pharmaceutical Drug. Available from: https://www.scribd.com/document/323179495/IMSH-Institute-Global-Oncology-Trend-2015-2020-Report

3. Evaluate Pharma. World Preview 2019, Outlook to 2024 [Internet]. [cited 2023 May 24]. Available from: https://info.evaluate.com/rs/607-YGS-364/images/EvaluatePharma_World_Preview_2019.pdf

4. Canadian Institute for Health Information. Prescribed Drug Spending in Canada. Canadian Institute for Health Information; 2020.

5. OECD. Pharmaceutical spending [Internet]. 2023 [cited 2021 Dec 1]. Available from: https://data.oecd.org/healthres/pharmaceutical-spending.htm#indicator-chart

6. Meyers DE, Jenei K, Chisamore TM, Gyawali B. Evaluation of the Clinical Benefit of Cancer Drugs Submitted for Reimbursement Recommendation Decisions in Canada. JAMA Internal Medicine. 2021;181(4):499–508.

7. Jenei K, Meyers D, Gyawali B. Assessment of Price and Clinical Benefit of Cancer Drugs in Canada, 2011-2020. JAMA Network Open. 2023 Jan 31;6(1):e2253438.

8. Cohen D. Cancer drugs: high price, uncertain value. BMJ (Clinical research ed). 2017;359:j4543.

9. Haycox A. Why Cancer? Pharmacoeconomics. 2016;34:625–7.

10. Godman B, Hill A, Simoens S, Selke G, Selke Krulichová I, Zampirolli Dias C, et al. Potential approaches for the pricing of cancer medicines across Europe to enhance the sustainability of healthcare systems and the implications. Expert Rev Pharmacoecon Outcomes Res. 2021 Aug;21(4):527–40.

11. 2019 [Internet]. [cited 2019 Aug 20]. About the pan-Canadian Oncology Drug Review (pCODR) | CADTH.ca. Available from: https://cadth.ca/pcodr/about-pcodr

12. CADTH. 2019. [cited 2019 Jul 17]. About CADTH. Available from: https://www.cadth.ca/about-cadth

13. Health CA for D and T in. pCODR Expert Review Committee Deliberative Framework [Internet]. 2016 [cited 2019 Oct 30] p. 1–7. Available from: www.cadth.ca/pcodr

For peer review only - http://bmjopen.bmj.com/site/about/guidelines.xhtml

14. Gamble JM, Weir DL, Johnson JA, Eurich DT. Analysis of drug coverage before and after the implementation of Canada's Common Drug Review. CMAJ. 2011 Nov 22;183(17):E1259.

15. Lexchin J. Financial conflicts of interest of clinicians making submissions to the pan-Canadian Oncology Drug Review: a descriptive study. BMJ Open. 2019;9:30750.

16. Meyers DE, Chisamore TM, McInnes MDF, Gyawali B, Prasad V, Booth CM. Industry payments to US physicians for cancer therapeutics: An analysis of the 2016–2018 open payments datasets. Journal of Cancer Policy. 2021 Jun 1;28:100283.

17. Mitchell AP, Trivedi NU, Gennarelli RL, Chimonas S, Tabatabai SM, Goldberg J, et al. Are Financial Payments from the Pharmaceutical Industry Associated with Physician Prescribing? A Systematic Review. Annals of Internal Medicine. 2021 Mar;174(3):353–61.

18. Cherny NI, Dafni U, Bogaerts J, Latino NJ, Pentheroudakis G, Douillard JY, et al. ESMO-Magnitude of Clinical Benefit Scale version 1.1. Annals of Oncology. 2017 Oct 1;28(10):2340–66.

19. Lexchin J. Canadian status of "drugs to avoid" in 2017: a descriptive analysis. CMAJ Open. 2018 Jul;6(3):E430.

20. Fojo T, Mailankody S, Lo A. Unintended consequences of expensive cancer therapeutics - The pursuit of marginal indications and a me-too mentality that stifles innovation and creativity: The John Conley lecture. JAMA Otolaryngology - Head and Neck Surgery. 2014;140(12):1225–36.

21. Cherla A, Naci H, Kesselheim AS, Gyawali B, Mossialos E. Assessment of Coverage in England of Cancer Drugs Qualifying for US Food and Drug Administration Accelerated Approval. JAMA Internal Medicine. 2021 Apr 1;181(4):490–8.

22. Kiesewetter B, Dafni U, Vries EGE de, Barriuso J, Curigliano G, González-Calle V, et al. ESMO-Magnitude of Clinical Benefit Scale for haematological malignancies (ESMO-MCBS:H) version 1.0. Annals of Oncology [Internet]. 2023 Jun 19 [cited 2023 Aug 4];0(0). Available from: https://www.annalsofoncology.org/article/S0923-7534(23)00729-9/fulltext

23. Jenei K, Raymakers AJN, Bayle A, Berger-Thürmel K, Cherla A, Honda K, et al. Health technology assessment for cancer medicines across the G7 countries and Oceania: an international, cross-sectional study. The Lancet Oncology. 2023 Jun 1;24(6):624–35.

24. Wild C, Grössmann N, Bonanno PV, Bucsics A, Furst J, Garuoliene K, et al. Utilisation of the ESMO-MCBS in practice of HTA. Ann Oncol. 2016 Nov;27(11):2134–6.

For peer review only

For peer review only - http://bmjopen.bmj.com/site/about/guidelines.xhtml

Table 1. Characteristics of clinicians that submitted input to the pan-Canadian Oncology Drug Review

**Characteristics of individual clinicians consulted**	
Total number of clinician feedback submissions	342 (100)
Unique clinicians	129 (100)
Number of funding decisions consulted	
One	76 (59)
Two	16 (12)
3 to 10	33 (26)
More than 10	4 (3)
Affiliation	
Physician	124 (96)
Oncology pharmacist	5 (4)
**Characteristic of clinician group submissions**	
Reimbursement decisions (2016-2020)	62 (100)
Reimbursement decisions with clinician submissions[a]	48 (100)
Clinician recommendation	
Fund	46 (96)
Do not fund	2 (4)
Median number of clinicians that offered feedback per drug review (IQR)	4 (2-8)

[a]Study sample

For peer review only

Table 2. Clinician group funding recommendation and supporting evidence

	Clinician group recommendation (n=48)	
	Fund (n=46)	Do not fund (n=2)
CADTH decision		
Positive	29 (63)	0 (0)
Negative	12 (26)	2 (100)
Submission w/conflicts		
Yes	44 (96)	1 (50)
No	2 (4)	1 (50)
Submissions w/ conflicts with manufacturer		
Yes	34 (74)	1 (50)
No	12 (26)	1 (50)
ESMO-MCBS Score		
Substantial benefit	23 (50)	2 (100)
No substantial benefit	23 (50)	0 (0)
RCT		
Yes	34 (74)	2 (100)
No	12 (26)	0 (0)
Phase		
1	3 (7)	0 (0)
2	10 (22)	0 (0)
3	33 (72)	2 (100)
Masked studies		
Yes	20 (43)	2 (100)
No	14 (30)	0 (0)
NR	12 (26)	0 (0)
OS		
Improved	18 (39)	0 (0)
Not improved	12 (26)	1 (50)
NA	16 (35)	1 (50)
PFS Data		
Improved	25 (54)	0 (0)
Not improved	5 (11)	0 (0)
NA	16 (35)	2 (100)
HRQoL		
Improved	5 (11)	0 (0)
No change	32 (70)	2 (100)
Worsen	1 (2)	0 (0)
NA	5 (11)	0 (0)
Price (per 28 cycle)		
Under $5000	5 (11)	0 (0)
$5000 to $9,999	27 (59)	1 (50)
Over $10,000	14 (30)	1 (50)

For peer review only

Table 3. Financial conflict of interests among clinicians consulted on drug funding decisions

Characteristic	Submissions (n=342)
Clinician submissions declaring conflicts	228 (67)
Clinician submissions with conflicts with the manufacturer of the drug under review	149 (44)
Average number of conflicts per submission	3 (2-6)
Types of conflicts reported	
Advisory role	166 (51)
Honoraria	74 (23)
Conference	22 (7)
Research	22 (7)
Events	23 (7)
Operations	2 (1)
Travel	3 (1)
Speaking	12 (1)
Incomplete COI documentation	9 (3)

Table 4. Association between the number of clinicians declaring financial conflicts of interests and supporting evidence for positive group recommendations[a]

	FCOI status			
Clinical gains	Conflict with submission	Conflict with another company	No conflict	p-value
ESMO-MCBS Score				
Substantial benefit	84	34	45	0.10
No substantial benefit	65	34	60	
OS benefit				
Improved	56	31	38	0.29
Not improved	40	12	25	
HRQoL status				
Improved	26	12	22	0.77
Worsened	12	4	12	

[a]FCOI status is defined as the number of clinicians declaring a financial conflict of interest; positive clinician funding recommendations include partial agreement with positive CADTH recommendation; total values differ between supporting evidence categories due to data availability of the clinical gain; association between FCOI status and clinical gains was analysed using Fisher's exact test.

For peer review only

For peer review only - http://bmjopen.bmj.com/site/about/guidelines.xhtml

Figure 1. Proportion of clinicians reporting conflicts per individual submission (n=46)

aTwo submissions were removed from the figure due to incomplete information

For peer review only

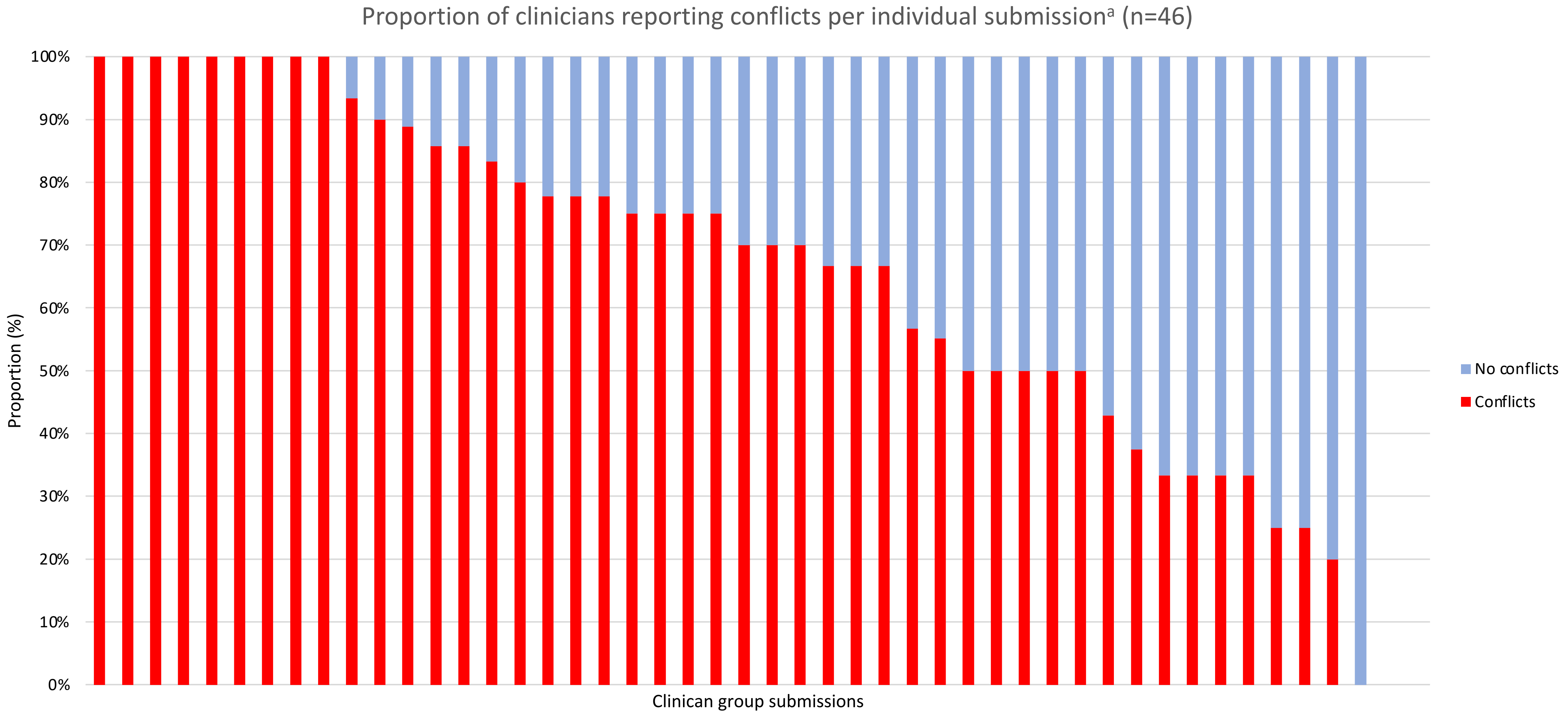

[a]Two submissions were removed from the figure due to incomplete information

STROBE Statement—Checklist of items that should be included in reports of ***cohort studies***

Characteristics of clinician input in Canadian funding decisions for cancer drugs

	Item No	Recommendation	Page No
**Title and abstract**	1	(*a*) Indicate the study's design with a commonly used term in the title or the abstract<br>(*b*) Provide in the abstract an informative and balanced summary of what was done and what was found	1-2
**Introduction**			
Background/rationale	2	Explain the scientific background and rationale for the investigation being reported	5
Objectives	3	State specific objectives, including any prespecified hypotheses	5
**Methods**			
Study design	4	Present key elements of study design early in the paper	6
Setting	5	Describe the setting, locations, and relevant dates, including periods of recruitment, exposure, follow-up, and data collection	6
Participants	6	(*a*) Give the eligibility criteria, and the sources and methods of selection of participants. Describe methods of follow-up<br>(*b*) For matched studies, give matching criteria and number of exposed and unexposed	N/A
Variables	7	Clearly define all outcomes, exposures, predictors, potential confounders, and effect modifiers. Give diagnostic criteria, if applicable	6
Data sources/ measurement	8*	For each variable of interest, give sources of data and details of methods of assessment (measurement). Describe comparability of assessment methods if there is more than one group	6
Bias	9	Describe any efforts to address potential sources of bias	NA
Study size	10	Explain how the study size was arrived at	6
Quantitative variables	11	Explain how quantitative variables were handled in the analyses. If applicable, describe which groupings were chosen and why	6
Statistical methods	12	(*a*) Describe all statistical methods, including those used to control for confounding<br>(*b*) Describe any methods used to examine subgroups and interactions<br>(*c*) Explain how missing data were addressed<br>(*d*) If applicable, explain how loss to follow-up was addressed<br>(*e*) Describe any sensitivity analyses	NA (descriptive Page 6)
**Results**			
Participants	13*	(a) Report numbers of individuals at each stage of study—eg numbers potentially eligible, examined for eligibility, confirmed eligible, included in the study, completing follow-up, and analysed<br>(b) Give reasons for non-participation at each stage<br>(c) Consider use of a flow diagram	8
Descriptive data	14*	(a) Give characteristics of study participants (eg demographic, clinical, social) and information on exposures and potential confounders<br>(b) Indicate number of participants with missing data for each variable of interest	8

For peer review only - http://bmjopen.bmj.com/site/about/guidelines.xhtml

		(c) Summarise follow-up time (eg, average and total amount)	
Outcome data	15*	Report numbers of outcome events or summary measures over time	8

For peer review only

Main results	16	(*a*) Give unadjusted estimates and, if applicable, confounder-adjusted estimates and their precision (eg, 95% confidence interval). Make clear which confounders were adjusted for and why they were included<br>(*b*) Report category boundaries when continuous variables were categorized<br>(*c*) If relevant, consider translating estimates of relative risk into absolute risk for a meaningful time period	8
Other analyses	17	Report other analyses done—eg analyses of subgroups and interactions, and sensitivity analyses	NA
**Discussion**			
Key results	18	Summarise key results with reference to study objectives	
Limitations	19	Discuss limitations of the study, taking into account sources of potential bias or imprecision. Discuss both direction and magnitude of any potential bias	9
Interpretation	20	Give a cautious overall interpretation of results considering objectives, limitations, multiplicity of analyses, results from similar studies, and other relevant evidence	9
Generalisability	21	Discuss the generalisability (external validity) of the study results	9/10
**Other information**			
Funding	22	Give the source of funding and the role of the funders for the present study and, if applicable, for the original study on which the present article is based	1

*Give information separately for exposed and unexposed groups.

Note: An Explanation and Elaboration article discusses each checklist item and gives methodological background and published examples of transparent reporting. The STROBE checklist is best used in conjunction with this article (freely available on the Web sites of PLoS Medicine at http://www.plosmedicine.org/, Annals of Internal Medicine at http://www.annals.org/, and Epidemiology at http://www.epidem.com/). Information on the STROBE Initiative is available at http://www.strobe-statement.org.

For peer review only

For peer review only - http://bmjopen.bmj.com/site/about/guidelines.xhtml

BMJ Open

Characteristics of clinician input in Canadian funding decisions for cancer drugs: a cross-sectional study based on CADTH reimbursement recommendations

Journal:	*BMJ Open*
Manuscript ID	bmjopen-2022-066378.R3
Article Type:	Original research
Date Submitted by the Author:	26-Aug-2023
Complete List of Authors:	Jenei, Kristina ; The London School of Economics and Political Science, Department of Health Policy<br>Meyers, Daniel E.; University of Calgary, Department of Medicine
**Primary Subject Heading**:	Health economics
Secondary Subject Heading:	Oncology, Evidence based practice
Keywords:	Health policy < HEALTH SERVICES ADMINISTRATION & MANAGEMENT, HEALTH ECONOMICS, ONCOLOGY

SCHOLARONE™
Manuscripts

I, the Submitting Author has the right to grant and does grant on behalf of all authors of the Work (as defined in the below author licence), an exclusive licence and/or a non-exclusive licence for contributions from authors who are: i) UK Crown employees; ii) where BMJ has agreed a CC-BY licence shall apply, and/or iii) in accordance with the terms applicable for US Federal Government officers or employees acting as part of their official duties; on a worldwide, perpetual, irrevocable, royalty-free basis to BMJ Publishing Group Ltd ("BMJ") its licensees and where the relevant Journal is co-owned by BMJ to the co-owners of the Journal, to publish the Work in this journal and any other BMJ products and to exploit all rights, as set out in our licence.

The Submitting Author accepts and understands that any supply made under these terms is made by BMJ to the Submitting Author unless you are acting as an employee on behalf of your employer or a postgraduate student of an affiliated institution which is paying any applicable article publishing charge ("APC") for Open Access articles. Where the Submitting Author wishes to make the Work available on an Open Access basis (and intends to pay the relevant APC), the terms of reuse of such Open Access shall be governed by a Creative Commons licence – details of these licences and which Creative Commons licence will apply to this Work are set out in our licence referred to above.

Other than as permitted in any relevant BMJ Author's Self Archiving Policies, I confirm this Work has not been accepted for publication elsewhere, is not being considered for publication elsewhere and does not duplicate material already published. I confirm all authors consent to publication of this Work and authorise the granting of this licence.

For peer review only

Characteristics of clinician input in Canadian funding decisions for cancer drugs: a cross-sectional study based on CADTH reimbursement recommendations

Kristina Jenei BSN MSc[1] & Daniel E. Meyers MD MSc[2],

[1] London School of Economics and Political Science
[2] Department of Medicine, University of Calgary, Calgary, Canada

Word count (text): 2990
Tables: 4
Figure: 1

Corresponding author:
Kristina Jenei, BSN MSc
Address: Houghton Street
London, United Kingdom
WC2A 2AE Telephone: +44-7911-494-962
Email: k.jenei@lse.ac.uk

For peer review only

Structured Abstract

Objective: To examine characteristics of clinician input to the pan-Canadian Oncology Drug Review (pCODR) for cancer drug funding recommendations from 2016 to 2020.

Design, Setting, and Participants: Descriptive, cross-sectional study included 62 reimbursement decisions from pCODR from 2016 to 2020.

Interventions: pCODR recommendations were analyzed for the number of clinicians consulted on each submission, affiliation, number of submissions per clinician, declared financial conflict of interests (FCOIs), randomization, type of masking, primary endpoint, study phase, and whether the study demonstrated improvement in overall survival (OS) and progression free survival (PFS).

Main outcome measures: The main outcome was clinician support for the initial funding recommendation. Secondary outcome measure was the association between clinician FCOI and clinical benefit in positive recommendations.

Results: The study consisted of 62 submissions, in which 48 included clinician input. A total of 129 unique clinicians provided 342 consultations. The majority (59%) provided input on less than five submissions, however, a small proportion (4%) consulted on over 10. Nearly all clinicians were physicians (125; 96%). From the 342 consultations, 228 declared financial conflicts (67%). The most common conflicts were payments for advisory roles (51%) and honorariums (23%). Of the 48 cancer drugs under review, clinicians recommended funding 46 (96%). Only 12 (25%) demonstrated substantial benefit, according to the ESMO-MCBS score. Drugs recommended for funding were more likely to have improved PFS and OS data. However, most cancer drugs supported by clinicians demonstrated no change in health-related quality of life (HRQoL), including one that demonstrated worsened HRQoL. There was no statistically significant difference between FCOI status and recommending drugs with health gains.

Conclusion: Clinicians offer crucial information in funding decisions. However, we found clinicians strongly supported funding nearly all cancer drugs under review, despite most not offering substantial benefit to patients nor gains in quality of life. While these drugs might be helpful options in clinical practice, funding numerous cancer drugs may be unsustainable for public health systems.

For peer review only

For peer review only - http://bmjopen.bmj.com/site/about/guidelines.xhtml

Strengths and Limitations

- The study examines data from CADTH reimbursement recommendations that are used to guide provincial funding decisions in Canadian provinces
- To the best of our knowledge, this study is the first to examine characteristics of clinician group input with the quality of evidence that underpin the drugs in Canadian funding decisions
- While the study uses the ESMO-MCBS tool to assess clinical value, the cohort examined is a homogenous sample of solid tumour indications which, cannot be generalised to all malignancies

For peer review only

Keywords
Health technology assessment; oncology; drug approvals; health policy; conflict of interest

For peer review only

For peer review only - http://bmjopen.bmj.com/site/about/guidelines.xhtml

Introduction

Country expenditure on cancer drugs has risen worldwide. Oncology medicines often account for the largest proportion of drug expenditure. Further, the costs of oncology service delivery have grown. In Europe, cancer care accounts for 30% of total hospital expenditure across Europe.(1) Indeed, sales of oncology medicines are expected to grow to $237 billion by 2024, with many new drugs under development.(2,3) In Canada, the largest proportion of Canadian public drug funding (22.9%) was spent on oncology agents.(4) Indeed, Canada is third highest for total dollars spent on pharmaceuticals among 31 countries included in The Organisation for Economic Co-operation and Development (OECD).(5) The trend of rising costs is concerning due to uncertain or negligible health gain often offered by new therapeutics.(6) Furthermore, previous research has found weak correlation between overall survival and the prices of new medicines at market launch.(7) Taken together, these challenges threaten the sustainability of publicly funded healthcare systems globally.(8–10)

Many countries with publicly funded health care systems employ health technology assessments to evaluate the value of new health technologies. These assessments often include stakeholder perspectives to ensure values perspectives are considered in the funding decision. In Canada, the Canadian Agency for Drugs and Technology in Health (CADTH) is the national health technology assessment (HTA) body that guides funding recommendations for Canadian provinces. CADTH, and its oncology assessment arm, the pan-Canadian Oncology Drug Review (pCODR)(11), evaluates medicines through a deliberative framework that includes clinical and economic evidence, clinician, and patient feedback.(12) While there are special authorisations, generally only medicines that are funded by the province are accessible to patients. Once a drug is reviewed, the pCODR Expert Review Committee (pERC)(13) — an appointed interdisciplinary body comprised of clinicians, ethicists, health economists, and patients — provide the final reimbursement recommendation. While each Canadian province independently decides whether a drug will be funded under their public drug plans, provinces often follow CADTH recommendations.(14) Since 2021, the pCODR and Common Drug Review (HTA assessment all other health interventions) has been aligned within one process. However, the responsibility of recommendations to pERC and the process for clinician group feedback remains the same.

Clinicians are involved in Canadian cancer drug funding decisions through two mechanisms. First, every drug review team includes oncologists to develop the systematic review protocol, interpret and provide real-world applicability for the study results. Once pERC reviews the clinical and economic reports and provides an initial funding recommendation, there is a second opportunity for clinician feedback. Since October 2016, CADTH provides an opportunity for additional feedback through external “calls for clinician input”. Registered clinicians, such as oncologists, pharmacists, and oncology nurses can submit feedback. While submissions can be made individually, CADTH encourages clinicians to submit in a group or health care association. Each submission must include an oncologist. This feedback is used to determine the final funding recommendation and published on the pCODR website.

For peer review only - http://bmjopen.bmj.com/site/about/guidelines.xhtml

Given trial participants are often non-representative of clinical practice, clinician and patient perspectives are important to prioritise to understand the value of medicine in real world settings. However, previous research exploring the role of clinicians in Canadian funding decisions found widespread financial conflict of interests (FCOIs).(15) In the United States (US), FCOIs are widespread in oncology(16) and have been found to influence prescribing patterns, formulary recommendations, research, and recommendations for clinical practice guidelines.(17) Given these associations, we sought to evaluate characteristics of clinician input beyond FCOIs, including the alignment between funding support and underlying clinical evidence for the cancer drugs under review.

For peer review only

Methods

We conducted a cross-sectional, descriptive study of reimbursement recommendations to pCODR for solid tumour indications between 2016 and 2020. The study examined publicly available data and therefore was exempt from institutional review board approval, in accordance with the US Department of Health and Human Services, 45 CFR §46.102(f). We adhered to the Strengthening the Reporting of Observational studies in Epidemiology (STROBE) reporting guidelines.

Source of data

A search of the pCODR website was completed to identify characteristics of physician submissions and supporting evidence. We used a sample of solid tumor indications from an existing dataset.(6) Supportive care medicines, hematologic neoplasms, pediatric indications, and biosimilars were excluded. By focusing on solid tumor indications, the dataset was homogenous which allowed us to assess the clinical benefit using the European Society for Medical Oncology Magnitude of Clinical Benefit Scale (ESMO-MCBS)(18), which was designed for solid tumour indications. We extracted the name of the drug, date and year of reimbursement recommendation and supporting clinical trial information, such as randomization, masking, overall survival and progression free survival outcomes, health related quality of life data, and estimated price (per 28-day cycle). While final drug prices are confidentially negotiated with the manufacturer after the funding recommendation, estimated prices are included in the pCODR economic evaluation reports. For more information on how these data were collected and how the ESMO-MCBS scores were calculated, see the previous publication.(6)

Clinician input on cancer drug submissions

In addition to the clinical evidence supporting each drug, we extracted data related to the clinician submissions. These data included the number of clinicians consulted on each submission, affiliation, number of submissions per clinician, and declared FCOIs. Clinicians provide feedback on the initial funding recommendation. On the submission forms, clinicians are asked whether they “agree”, “disagree”, or “agree in part” with the initial funding recommendation. With this feedback, pCODR can revise the final funding recommendation. While there are often numerous clinicians involved in each drug review, the feedback form is submitted collectively. When the clinicians agreed with a positive funding recommendation or disagreed with a negative funding recommendation, we coded this as “fund”. When the clinicians agreed with a negative recommendation or disagreed with a positive funding recommendation, we coded this as “do not fund”. When the clinician feedback was to “agree in part”, we reviewed comments in the feedback documents to ensure coding was accurate.

Conflict of interest data

Despite one collective feedback form, FCOI forms are submitted individually. FCOI forms require clinicians to disclose whether they have received payments over the previous two years from any company or organization that may have direct or indirect interest in the drug under review. The forms require disclosure on the type of activity the payment was for (e.g., advisory board, conferences, honoraria, research, sponsorship

For peer review only

of events, operations, travel, among others). Clinicians must also provide the names of the manufacturers who they have received funds from, including whether these payments exceeded CAD$10,000 and whether they have personal or commercial relationships with any interest group in relation to the drug under submission. Information related to all these categories were also extracted. We then investigated the association between FCOI status (conflict with submission, conflict with other company, no conflict) and clinical benefit for drugs that received positive recommendations to understand whether FCOI would impact funding recommendations.

Data analysis

Descriptive data and counts are presented throughout this manuscript. While FCOI documentation is submitted individually, clinician feedback is sought through groups and cannot be disaggregated further. Therefore, due to CADTH procedures, we analysed clinician group submissions. Financial conflicts of interest were analysed as individuals given available data. We explored the association of FCOI status (conflict with the company, conflict with another company, no conflict) in recommending drugs with meaningful benefit (defined as improvements in ESMO-MCBS score, OS benefit, and HRQoL status). The purpose of this analysis was to understand the role of FCOIs in recommending drugs with or without health gains. Data per submission were aggregated to compare these factors. Both analyses used Fisher's exact test. Data analysis was conducted using Microsoft Excel (version 16.73) and R Statistical Software (version 2023.06.1+524).

Patient and Public Involvement

No patients nor publics were involved.

For peer review only

Results

Table 1 reports the descriptive characteristics of clinicians that were consulted during cancer drug funding decisions. Overall, there were 62 drug funding reviews by CADTH for solid tumour indications between 2016 and 2020. Of these, clinician group input were available for 48 submissions. For 14, there were no clinician feedback published on the pCODR website (there are no reasons provided for why these submissions did not have clinician input). On average, 4 (IQR: 2-8) clinicians provided feedback per drug. In the 48 drug reviews, there was 342 submissions from clinicians for feedback on funding recommendations. However, there were only 129 unique clinicians which suggests clinicians often consulted on more than one drug. Of these, 124 (96%) were physicians and 5 (4%) were oncology pharmacists. Most clinicians (76; 59%) consulted on only one drug review. However, a small minority (4; 3%) made repeat submissions on more than 10 drugs.

Table 2 reports the clinician recommendations and supporting evidence. Of the 48 submissions that included clinician input, 46 (96%) had positive clinician feedback to fund the drug. This included 12 (26%) drugs that received negative recommendations from CADTH in which clinicians submitted feedback in support of funding the drug. Only 2 (4%) submissions believed the drug should not be funded.
Of the drugs recommended for funding, most (32; 70%) did not offer substantial benefit, as per ESMO-MCBS.

In terms of clinical benefit, clinicians recommended funding 18 (39%) drugs that had data that demonstrated an OS benefit, 12 (26%) without an OS benefit, and 16 (35%) that had no data on OS. Similarly, 25 drugs (54%) that demonstrated an improved PFS benefit, 5 (11%) that did not improve PFS, and 16 drugs (35%) that did not have any data related to PFS received funding support from clinicians. Nearly two thirds of drugs (32; 70%) that received funding support by clinicians demonstrated no change in HRQoL, with one that worsened HRQoL outcomes. Most cancer drugs that received clinician funding support were between $5000 to $9,999 per month (59%), including 14 (30%) that were over $10,000 per 28-day cycle (Table 3).

We found numerous financial conflicts of interests **(Figure 1)**. Of the 48 submissions made by clinician groups, 46 had complete FCOI disclosures that were able to be analysed (9 of 342 individual documents; 3%) were incomplete. Of the 342 instances where clinicians were consulted during the drug review, 228 (67%) reported FCOIs **(Table 3)**. Of these, 149 (44%) were with the manufacturer of the drug under review. The average number of conflicts a clinician disclosed was 3 (IQR: 2 to 6). Advisory roles were the most common type of conflict (166; 51%), followed by honorariums (23%), payments to attend conferences, research, and sponsorship of events (7%, respectively). We found 9 documents (3%) that were incomplete.
We extended this analysis to understand the role of FCOIs in recommending drugs with or without meaningful clinical benefit in positive clinician group recommendations (**Table 4).** We found that there was no statistically significant difference between clinician FCOI status and clinical benefit per ESMO-MCBS score, overall survival benefit, nor improvements with health-related quality of life.

For peer review only - http://bmjopen.bmj.com/site/about/guidelines.xhtml

For peer review only

Discussion

We sought to examine characteristics of clinicians consulted in Canadian cancer drug funding decisions from 2016 to 2020. We examined clinician recommendations and supporting evidence for the cancer drugs under review for reimbursement. Further, we explored the extent of FCOIs disclosed in clinician submissions and their association with recommending drugs with meaningful clinical benefit. We found that clinicians who consult on Canadian funding decisions support funding the cancer drug under review. Of the 48 drugs, 46 (96%) received supportive funding feedback from clinicians, even when most drugs did not offer substantial benefit to patients nor improved quality of life. These findings have important implications.

First, our findings highlight a substantial proportion of new cancer medicines are introduced in the Canadian market with high prices and uncertain or negligible value. It is reasonable to assume many clinicians would prefer robust, mature evidence demonstrating improvements patient-cantered outcomes, such as improvements in overall survival and quality of life. However, in a previous study, we found that only half of cancer drugs that received a positive funding recommendation from pCODR had evidence of improved survival.(6) Similarly, the Patented Medicine Prices Review Board (PMPRB) —the federal agency responsible for regulating drug prices of new patented medicines in Canada — classified over 90% of new medicines as offering “moderate, little, or no improvement” in clinical benefit.(19) Therefore, in this ecosystem, objective and clear decision making is required. Influence from conflicts or vested interests might add strain to health budgets.

Second, our findings might highlight conflicting principal-agent relationships between national health technology assessment (HTA) bodies, pharmaceutical manufacturers, and clinicians. The duty of a clinician to care for individual patients differs from HTA bodies who attempt to maximize population health outcomes for every dollar spent. For clinicians, it might be advantageous to have numerous treatment options to offer patients, including a spectrum of available medicines given differential responses to individual therapies. However, at the national reimbursement level, funding numerous high-priced cancer drugs incurs a large budget impact. Indeed, 27 drugs (59%) that received positive funding support from clinicians were priced between $5000 to $9,999 per month. This is concerning in oncology where many high-priced cancer drugs approved through regulatory agencies often only offer marginal benefit, if any at all.(6,20,21)

Third, we found numerous conflicts of interests. Of the 342 submissions, 228 (67%) clinicians declared conflicts and nearly half were directly with the manufacturer of the drug under review. Clinicians that declared FCOIs with the manufacturer of the submission were more likely to recommend funding the drug. However, there was no statistically significant differences between the distribution of individual clinician FCOIs and recommendation for drugs with meaningful clinical benefit. This is an interesting finding that warrants further investigation to understand whether a true cause exists but may be related to the previously discussed desire to have numerous options for patients

For peer review only - http://bmjopen.bmj.com/site/about/guidelines.xhtml

given treatment responses that may differ from the clinical trial results. Given the overwhelming support for nearly all drugs in our sample, this finding may be skewed. Future research might repeat this study with the inclusion of haematological malignancies given the recently released ESMO-MCBS for this population.(22) Our results are aligned with previous Canadian research. Lexchin found that 75% of clinicians who disagreed with a negative funding recommendation declared a conflict with the manufacturer under review.(15) However, in the nine negative initial funding recommendations, pCODR changed their final recommendation once. Similarly, in our study, the initial funding recommendation was only changed once after stakeholder feedback. Further, we found 9 of 342 (3%) FCOI documents that were incomplete which raises concerns about the disclosure process. The literature about FCOIs and clinician behaviour suggests that there is a relationship between financial payments to clinicians and prescribing patterns, formulary recommendations, research, and recommendations for clinical practice guidelines.(16,17)

For peer review only

Our findings raise concerns about the use of stakeholder perspectives in funding decisions and are relevant to other countries outside of Canada that use similar HTA methodology. Countries, such as England, Italy, New Zealand, Australia, among others, include input from a variety of external groups in funding decisions.(23) However, Canada is the only country that includes patient and clinician group feedback in every decision. Other countries include stakeholder input as deemed necessary, such as funding a drug for a rare or severe disease. While CADTH includes patient and clinician group feedback to ensure fair decisions, the funding recommendations from these groups are complicated by numerous FCOIs and overwhelmingly positive support for the drug. Furthermore, we found several clinicians that consult on decisions more than once, with a small proportion consulting on over 10. Repeat consultations may be warranted by the nature of oncology, where novel therapeutics treat small, specialized populations, similar to those of rare diseases. To manage provider-induced demand, CADTH may want to reconsider how the agency engages with clinicians, or whether the agency ought to incorporate external stakeholder feedback on every funding decision, given overwhelming support for funding nearly every drug independent of clinical benefit. Furthermore, CADTH, among other HTA bodies, may want to be more explicit for how stakeholder input is used in funding decisions.

Strengths and Limitations

Our retrospective cohort study has strengths and limitations. It is unique as it is the first evaluation of characteristics of clinician input in Canadian funding decision that includes an assessment of the supporting evidence of the drugs under review. However, we encountered limitations. First, we were limited to a cohort of cancer drugs that was accepted and reviewed by CADTH. Second, feedback forms where clinicians indicate their support for drug funding are submitted collectively, not individually. Therefore, clinician input cannot be attributed to individual views. However, we were able to examine FCOI status (submitted individually) and the clinical benefit status of the recommended drug. Third, we are limited by a cohort of submissions for solid tumour indications which might differ compared to other malignancies. However, this approach allows for homogenous dataset for which clinical benefit can be measured using the

For peer review only - http://bmjopen.bmj.com/site/about/guidelines.xhtml

ESMO-MCBS. Further, several methodological limitations have been discussed regarding the ESMO-MCBS tool(24) (use of the lower limit confidence interval, focus on primary endpoints even for studies that use unvalidated surrogate endpoints, use of QoL data, and no weighted arguments for values) which should be acknowledged given it is the measure of value within our study. Lastly, we are limited by the publicly available data in pCODR reports. Given the complexity of evaluating evidence from oncology clinical trials, these reports might not offer insight into the nuanced discussions that likely occurred throughout the pCODR deliberations. Future research might build on our study to further examine the effects of FCOIs on the CADTH decision with more sophisticated statistical methods or qualitative analysis.

Conclusions

Clinicians offer crucial information in funding decisions. However, we found overwhelming support for nearly all drugs under review, even for medicines that did not demonstrate improved OS benefit nor quality of life — two of the most important patients-centred outcomes. Further, we found numerous FCOIs. These findings raise questions about the role of stakeholder input in HTA funding decisions and possible incongruity between objectives of national health technology assessment bodies and practising clinicians.

For peer review only

For peer review only - http://bmjopen.bmj.com/site/about/guidelines.xhtml

Ethics approval: The study examined publicly available data and therefore was exempt from institutional review board approval.

Contributor statement: Ms. Jenei lead the analysis, drafting, and dissemination. Dr. Meyers were involved in the drafting and editing of the manuscript.

Competing interests: Ms. Jenei and Dr. Meyers report no conflicts.

Funding statement: Ms. Jenei is supported by a Canadian Institutes of Health Research Doctoral Foreign Study Award (181603). The funder was not involved in the manuscript.

Data sharing statement: Data are available upon reasonable request.

For peer review only

References

1. Simoens S, KU Leuven, Leuven, Belgium, Van Harten W, University Twente, Enschede, The Netherlands, Rijnstate Hospital, Arnhem, The Netherlands, Lopes G, et al. What Happens when the Cost of Cancer Care Becomes Unsustainable? European Oncology & Haematology. 2017;13(02):108.

2. Scribd [Internet]. [cited 2023 May 24]. IMSH Institute Global Oncology Trend 2015 2020 Report | PDF | Non Small Cell Lung Carcinoma | Pharmaceutical Drug. Available from: https://www.scribd.com/document/323179495/IMSH-Institute-Global-Oncology-Trend-2015-2020-Report

3. Evaluate Pharma. World Preview 2019, Outlook to 2024 [Internet]. [cited 2023 May 24]. Available from: https://info.evaluate.com/rs/607-YGS-364/images/EvaluatePharma_World_Preview_2019.pdf

4. Canadian Institute for Health Information. Prescribed Drug Spending in Canada. Canadian Institute for Health Information; 2020.

5. OECD. Pharmaceutical spending [Internet]. 2023 [cited 2021 Dec 1]. Available from: https://data.oecd.org/healthres/pharmaceutical-spending.htm#indicator-chart

6. Meyers DE, Jenei K, Chisamore TM, Gyawali B. Evaluation of the Clinical Benefit of Cancer Drugs Submitted for Reimbursement Recommendation Decisions in Canada. JAMA Internal Medicine. 2021;181(4):499–508.

7. Jenei K, Meyers D, Gyawali B. Assessment of Price and Clinical Benefit of Cancer Drugs in Canada, 2011-2020. JAMA Network Open. 2023 Jan 31;6(1):e2253438.

8. Cohen D. Cancer drugs: high price, uncertain value. BMJ (Clinical research ed). 2017;359:j4543.

9. Haycox A. Why Cancer? Pharmacoeconomics. 2016;34:625–7.

10. Godman B, Hill A, Simoens S, Selke G, Selke Krulichová I, Zampirolli Dias C, et al. Potential approaches for the pricing of cancer medicines across Europe to enhance the sustainability of healthcare systems and the implications. Expert Rev Pharmacoecon Outcomes Res. 2021 Aug;21(4):527–40.

11. 2019 [Internet]. [cited 2019 Aug 20]. About the pan-Canadian Oncology Drug Review (pCODR) | CADTH.ca. Available from: https://cadth.ca/pcodr/about-pcodr

12. CADTH. 2019. [cited 2019 Jul 17]. About CADTH. Available from: https://www.cadth.ca/about-cadth

13. Health CA for D and T in. pCODR Expert Review Committee Deliberative Framework [Internet]. 2016 [cited 2019 Oct 30] p. 1–7. Available from: www.cadth.ca/pcodr

For peer review only - http://bmjopen.bmj.com/site/about/guidelines.xhtml

14. Gamble JM, Weir DL, Johnson JA, Eurich DT. Analysis of drug coverage before and after the implementation of Canada's Common Drug Review. CMAJ. 2011 Nov 22;183(17):E1259.

15. Lexchin J. Financial conflicts of interest of clinicians making submissions to the pan-Canadian Oncology Drug Review: a descriptive study. BMJ Open. 2019;9:30750.

16. Meyers DE, Chisamore TM, McInnes MDF, Gyawali B, Prasad V, Booth CM. Industry payments to US physicians for cancer therapeutics: An analysis of the 2016–2018 open payments datasets. Journal of Cancer Policy. 2021 Jun 1;28:100283.

17. Mitchell AP, Trivedi NU, Gennarelli RL, Chimonas S, Tabatabai SM, Goldberg J, et al. Are Financial Payments from the Pharmaceutical Industry Associated with Physician Prescribing? A Systematic Review. Annals of Internal Medicine. 2021 Mar;174(3):353–61.

18. Cherny NI, Dafni U, Bogaerts J, Latino NJ, Pentheroudakis G, Douillard JY, et al. ESMO-Magnitude of Clinical Benefit Scale version 1.1. Annals of Oncology. 2017 Oct 1;28(10):2340–66.

19. Lexchin J. Canadian status of “drugs to avoid” in 2017: a descriptive analysis. CMAJ Open. 2018 Jul;6(3):E430.

20. Fojo T, Mailankody S, Lo A. Unintended consequences of expensive cancer therapeutics - The pursuit of marginal indications and a me-too mentality that stifles innovation and creativity: The John Conley lecture. JAMA Otolaryngology - Head and Neck Surgery. 2014;140(12):1225–36.

21. Cherla A, Naci H, Kesselheim AS, Gyawali B, Mossialos E. Assessment of Coverage in England of Cancer Drugs Qualifying for US Food and Drug Administration Accelerated Approval. JAMA Internal Medicine. 2021 Apr 1;181(4):490–8.

22. Kiesewetter B, Dafni U, Vries EGE de, Barriuso J, Curigliano G, González-Calle V, et al. ESMO-Magnitude of Clinical Benefit Scale for haematological malignancies (ESMO-MCBS:H) version 1.0. Annals of Oncology [Internet]. 2023 Jun 19 [cited 2023 Aug 4];0(0). Available from: https://www.annalsofoncology.org/article/S0923-7534(23)00729-9/fulltext

23. Jenei K, Raymakers AJN, Bayle A, Berger-Thürmel K, Cherla A, Honda K, et al. Health technology assessment for cancer medicines across the G7 countries and Oceania: an international, cross-sectional study. The Lancet Oncology. 2023 Jun 1;24(6):624–35.

24. Wild C, Grössmann N, Bonanno PV, Bucsics A, Furst J, Garuoliene K, et al. Utilisation of the ESMO-MCBS in practice of HTA. Ann Oncol. 2016 Nov;27(11):2134–6.

For peer review only - http://bmjopen.bmj.com/site/about/guidelines.xhtml

Table 1. Characteristics of clinicians that submitted input to the pan-Canadian Oncology

**Characteristics of individual clinicians consulted**	
Total number of clinician feedback submissions	342 (100)
Unique clinicians	129 (100)
Number of funding decisions consulted	
One	76 (59)
Two	16 (12)
3 to 10	33 (26)
More than 10	4 (3)
Affiliation	
Physician	124 (96)
Oncology pharmacist	5 (4)
**Characteristic of clinician group submissions**	
Reimbursement decisions (2016-2020)	62 (100)
Reimbursement decisions with clinician submissions[a]	48 (100)
Clinician recommendation	
Fund	46 (96)
Do not fund	2 (4)
Median number of clinicians that offered feedback per drug review (IQR)	4 (2-8)

Drug Review

[a]Study sample

For peer review only

476 **Table 2.** Clinician group funding recommendation and supporting evidence

	Clinician group recommendation (n=48)	
	Fund (n=46)	Do not fund (n=2)
CADTH decision		
Positive	29 (63)	0 (0)
Negative	12 (26)	2 (100)
Submission w/conflicts		
Yes	44 (96)	1 (50)
No	2 (4)	1 (50)
Submissions w/ conflicts with manufacturer		
Yes	34 (74)	1 (50)
No	12 (26)	1 (50)
ESMO-MCBS Score		
Substantial benefit	23 (50)	2 (100)
No substantial benefit	23 (50)	0 (0)
RCT		
Yes	34 (74)	2 (100)
No	12 (26)	0 (0)
Phase		
1	3 (7)	0 (0)
2	10 (22)	0 (0)
3	33 (72)	2 (100)
Masked studies		
Yes	20 (43)	2 (100)
No	14 (30)	0 (0)
NR	12 (26)	0 (0)
OS		
Improved	18 (39)	0 (0)
Not improved	12 (26)	1 (50)
NA	16 (35)	1 (50)
PFS Data		
Improved	25 (54)	0 (0)
Not improved	5 (11)	0 (0)
NA	16 (35)	2 (100)
HRQoL		
Improved	5 (11)	0 (0)
No change	32 (70)	2 (100)
Worsen	1 (2)	0 (0)
NA	5 (11)	0 (0)
Price (per 28 cycle)		
Under $5000	5 (11)	0 (0)
$5000 to $9,999	27 (59)	1 (50)
Over $10,000	14 (30)	1 (50)

477
478

For peer review only - http://bmjopen.bmj.com/site/about/guidelines.xhtml

Table 3. Financial conflict of interests among clinicians consulted on drug funding decisions

Characteristic	Submissions (n=342)
Clinician submissions declaring conflicts	228 (67)
Clinician submissions with conflicts with the manufacturer of the drug under review	149 (44)
Average number of conflicts per submission	3 (2-6)
Types of conflicts reported	
Advisory role	166 (51)
Honoraria	74 (23)
Conference	22 (7)
Research	22 (7)
Events	23 (7)
Operations	2 (1)
Travel	3 (1)
Speaking	12 (1)
Incomplete COI documentation	9 (3)

Table 4. Association between the number of clinicians declaring financial conflicts of interests and supporting evidence for positive group recommendations[a]

	FCOI status			
Clinical gains	Conflict with submission	Conflict with another company	No conflict	p-value
ESMO-MCBS Score				0.10
Substantial benefit	84	34	45	
No substantial benefit	65	34	60	
OS benefit				0.29
Improved	56	31	38	
Not improved	40	12	25	
HRQoL status				0.77
Improved	26	12	22	
Worsened	12	4	12	

[a]FCOI status is defined as the number of clinicians declaring a financial conflict of interest that participated in the drug submission process; positive clinician funding recommendations include partial agreement with positive CADTH recommendation; total values differ between supporting evidence

For peer review only

For peer review only - http://bmjopen.bmj.com/site/about/guidelines.xhtml

categories due to data availability of the clinical gain; association between FCOI status and clinical gains was analysed using Fisher's exact test.

Figure 1. Proportion of clinicians reporting conflicts per individual submission (n=46)

aTwo submissions were removed from the figure due to incomplete information

For peer review only

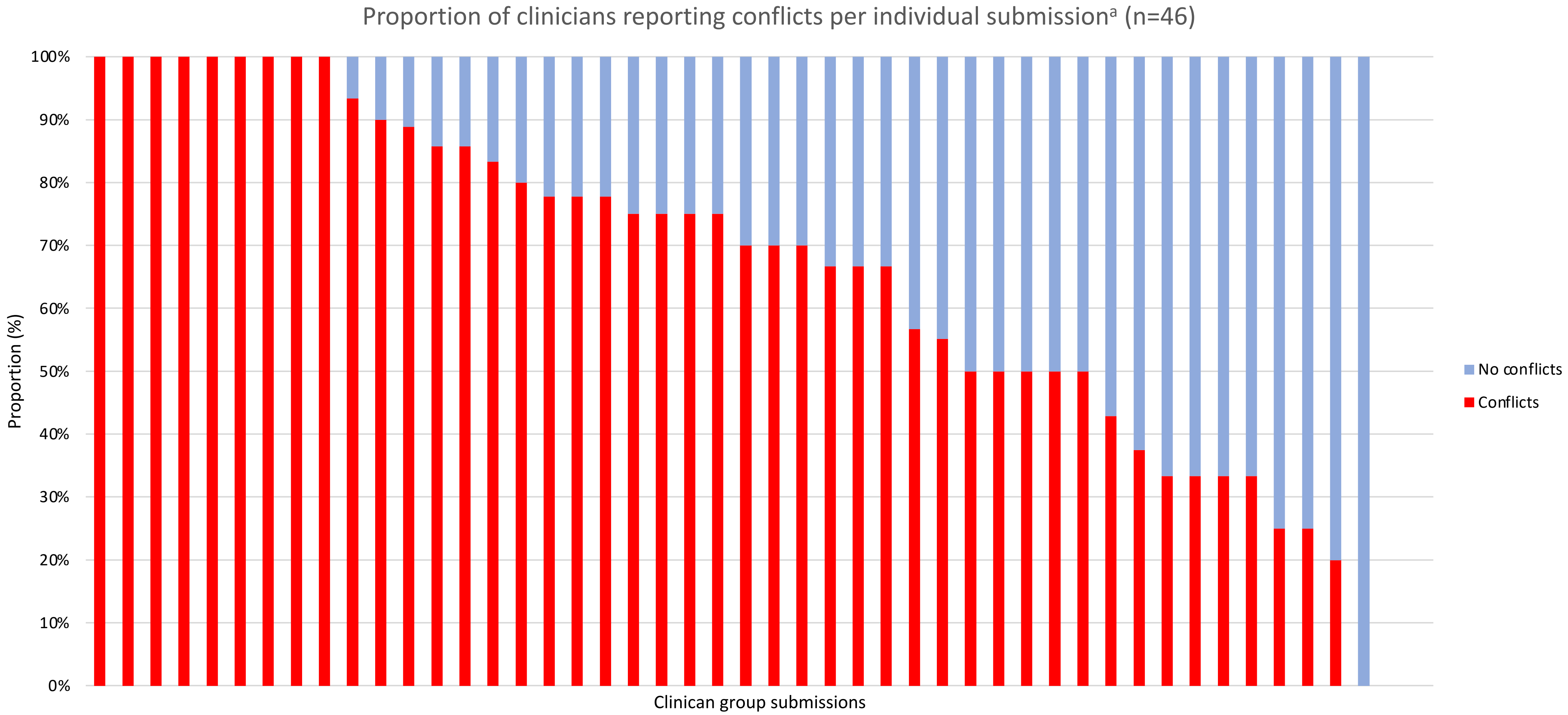

[a]Two submissions were removed from the figure due to incomplete information

STROBE Statement—Checklist of items that should be included in reports of ***cohort studies***

Characteristics of clinician input in Canadian funding decisions for cancer drugs: a cross-sectional study based on CADTH reimbursement recommendations

	Item No	Recommendation	Page No
**Title and abstract**	1	(*a*) Indicate the study's design with a commonly used term in the title or the abstract<br>(*b*) Provide in the abstract an informative and balanced summary of what was done and what was found	1-2
**Introduction**			
Background/rationale	2	Explain the scientific background and rationale for the investigation being reported	5-6
Objectives	3	State specific objectives, including any prespecified hypotheses	5-6
**Methods**			
Study design	4	Present key elements of study design early in the paper	7
Setting	5	Describe the setting, locations, and relevant dates, including periods of recruitment, exposure, follow-up, and data collection	7-8
Participants	6	(*a*) Give the eligibility criteria, and the sources and methods of selection of participants. Describe methods of follow-up<br>(*b*) For matched studies, give matching criteria and number of exposed and unexposed	N/A
Variables	7	Clearly define all outcomes, exposures, predictors, potential confounders, and effect modifiers. Give diagnostic criteria, if applicable	7-8
Data sources/ measurement	8*	For each variable of interest, give sources of data and details of methods of assessment (measurement). Describe comparability of assessment methods if there is more than one group	7
Bias	9	Describe any efforts to address potential sources of bias	NA
Study size	10	Explain how the study size was arrived at	7-8
Quantitative variables	11	Explain how quantitative variables were handled in the analyses. If applicable, describe which groupings were chosen and why	7-8
Statistical methods	12	(*a*) Describe all statistical methods, including those used to control for confounding<br>(*b*) Describe any methods used to examine subgroups and interactions<br>(*c*) Explain how missing data were addressed<br>(*d*) If applicable, explain how loss to follow-up was addressed<br>(*e*) Describe any sensitivity analyses	8
**Results**			
Participants	13*	(a) Report numbers of individuals at each stage of study—eg numbers potentially eligible, examined for eligibility, confirmed eligible, included in the study, completing follow-up, and analysed<br>(b) Give reasons for non-participation at each stage<br>(c) Consider use of a flow diagram	NA
Descriptive data	14*	(a) Give characteristics of study participants (eg demographic, clinical, social) and information on exposures and potential confounders<br>(b) Indicate number of participants with missing data for each variable of interest<br>(c) Summarise follow-up time (eg, average and total amount)	7-8

For peer review only - http://bmjopen.bmj.com/site/about/guidelines.xhtml

For peer review only

Outcome data	15*	Report numbers of outcome events or summary measures over time	8
Main results	16	(*a*) Give unadjusted estimates and, if applicable, confounder-adjusted estimates and their precision (eg, 95% confidence interval). Make clear which confounders were adjusted for and why they were included<br>(*b*) Report category boundaries when continuous variables were categorized<br>(*c*) If relevant, consider translating estimates of relative risk into absolute risk for a meaningful time period	9
Other analyses	17	Report other analyses done—eg analyses of subgroups and interactions, and sensitivity analyses	9-10
**Discussion**			
Key results	18	Summarise key results with reference to study objectives	11-12
Limitations	19	Discuss limitations of the study, taking into account sources of potential bias or imprecision. Discuss both direction and magnitude of any potential bias	12
Interpretation	20	Give a cautious overall interpretation of results considering objectives, limitations, multiplicity of analyses, results from similar studies, and other relevant evidence	11-12
Generalisability	21	Discuss the generalisability (external validity) of the study results	12
**Other information**			
Funding	22	Give the source of funding and the role of the funders for the present study and, if applicable, for the original study on which the present article is based	14

*Give information separately for exposed and unexposed groups.

Note: An Explanation and Elaboration article discusses each checklist item and gives methodological background and published examples of transparent reporting. The STROBE checklist is best used in conjunction with this article (freely available on the Web sites of PLoS Medicine at http://www.plosmedicine.org/, Annals of Internal Medicine at http://www.annals.org/, and Epidemiology at http://www.epidem.com/). Information on the STROBE Initiative is available at http://www.strobe-statement.org.

For peer review only
